# Phytochemical Analysis, Antioxidant and Bone Anabolic Effects of *Blainvillea acmella* (L.) Philipson

**DOI:** 10.3389/fphar.2021.796509

**Published:** 2022-01-17

**Authors:** Rohanizah Abdul Rahim, Putri Ayu Jayusman, Vuanghao Lim, Nor Hazwani Ahmad, Zuratul Ain Abdul Hamid, Sharlina Mohamed, Norliza Muhammad, Fairus Ahmad, Norfilza Mokhtar, Norazlina Mohamed, Ahmad Nazrun Shuid, Isa Naina Mohamed

**Affiliations:** ^1^ Pharmacology Department, Faculty of Medicine, Universiti Kebangsaan Malaysia, Kuala Lumpur, Malaysia; ^2^ Advanced Medical and Dental Institute, Universiti Sains Malaysia, Pulau Pinang, Malaysia; ^3^ Department of Craniofacial Diagnostics and Biosciences, Faculty of Dentistry, Universiti Kebangsaan Malaysia, Kuala Lumpur, Malaysia; ^4^ School of Materials and Mineral Resources Engineering, Universiti Sains Malaysia, Pulau Pinang, Malaysia; ^5^ Anatomy Department, Faculty of Medicine, Universiti Kebangsaan Malaysia, Kuala Lumpur, Malaysia; ^6^ Physiology Department, Faculty of Medicine, Universiti Kebangsaan Malaysia, Kuala Lumpur, Malaysia; ^7^ Faculty of Medicine UiTM, Sg Buloh, Malaysia

**Keywords:** Phytochemical, GCMS, LCMS, *Blainvillea acmella*, *Spilanthes acmella*, bone anabolic, osteoblast, antioxidant

## Abstract

*Blainvillea acmella* (L.) Philipson [Asteraceae] (*B. acmella*) is an important medicinal plant native to Brazil, and it is widely known as a toothache plant. A plethora of studies have demonstrated the antioxidant activities of *B. acmella* and few studies on the stimulatory effects on alkaline phosphatase (ALP) secretion from bone cells; however, there is no study on its antioxidant and anabolic activity on bone cells. The study aimed to evaluate the phytochemical contents of aqueous and ethanol extracts of *B. acmella* using gas chromatography mass spectrometry (GCMS) and liquid chromatography time of flight mass spectrometry (LCTOFMS) along with the total phenolic (TPC) and flavonoid (TFC) contents using Folin-Ciocalteu and aluminum colorimetric methods. The extracts of *B. acmella* leaves were used to scavenge synthetic-free radicals such as 2,2-diphenyl-1-picrylhydrazyl (DPPH), 2,2′-azino-bis-(3-ethylbenzothiazoline-6-sulfonic acid) (ABTS), and ferric reducing antioxidant power (FRAP) assays. The bone anabolic effects of *B. acmella* extracts on MC3T3-E1 cells were measured with 3-(4,5-dimethylthiazol-2-yl)-2, 5-diphenyltetrazoium bromide (MTT) at 1, 3, 5, and 7 days, Sirius-red and ALP at 7 and 14 days, and Alizarin Red S at 14 and 21 days. Comparatively, ethanol extract of *B. acmella* (*Ba*E) contributed higher antioxidant activities (IC_50_ of 476.71 µg/ml and 56.01 ± 6.46 mg L-ascorbic acid/g against DPPH and FRAP, respectively). Anabolic activities in bone proliferation, differentiation, and mineralization were also higher in *B. acmella* of ethanol (*Ba*E) than aqueous (*Ba*A) extracts. Positive correlations were observed between phenolic content (TPC and TFC) to antioxidant (ABTS and FRAP) and anabolic activities. Conversely, negative correlations were present between phenolic content to antioxidant (DPPH) activity. These potential antioxidant and bone anabolic activities in *Ba*E might be due to the phytochemicals confirmed through GCMS and LCTOFMS, revealed that terpenoids of α-cubebene, cryophyllene, cryophyllene oxide, phytol and flavonoids of pinostrobin and apigenin were the compounds contributing to both antioxidant and anabolic effects in *Ba*E. Thus, *B. acmella* may be a valuable antioxidant and anti-osteoporosis agent. Further study is needed to isolate, characterize and elucidate the underlying mechanisms responsible for the antioxidant and bone anabolic effects.

## Introduction

According to the World Health Organization (WHO) Global Atlas of Traditional, Complementary, and Alternative Medicine (Bodeker et al., 2005), traditional medicine has been used widely by the world population to treat diseases. Approximately 70–95% of the population in the developing world uses traditional medicine as primary care (Robinson and Zhang, 2011). The use of medicinal plants or isolated active compounds from plants has gained attention due to their perceived lower toxicity.

Osteoporosis is a chronic skeletal condition that is characterized by deterioration in bone microarchitectural and reduction in bone mass, leading to decreased bone strength and increased risk of bone fracture (Clynes et al., 2020). Reactive oxygen species (ROS) may inhibit the function of osteoblast cells in bone formation. This would lead to imbalances between bone formation and bone resorption, contributing to osteoporosis (Domazetovic et al., 2017). Current treatments such as calcitonin, estrogen, and bisphosphonates are anti-bone resorptive drugs that inhibit osteoclast activity (Yang et al., 2018). However, long-term use of these treatments is associated with several side effects, such as cancer, femur fractures, osteonecrosis of the jaw, myocardial infarction, thromboembolic events, and skin reaction (DRESS syndrome) (Khosla and Hofbauer, 2017; Pherwani and Sathaye, 2020).

Given the contraindications of anti-bone resorptive drugs, it is important to develop plant-based therapeutic agents that will stimulate proliferation and differentiation of osteoblastic cells in the treatment of osteoporosis with minimal side effects than conventional treatments (Martiniakova et al., 2020). The osteoblast is one of the bone cells that play a major role in bone formation for new bone development (Kenkre and Bassett, 2018). Osteoblast has been used widely as a model system to study bone differentiation and mineralization based on the resemblance between the *in vitro* osteogenic differentiation and *in vivo* bone formation (Owen et al., 1990; Quarles et al., 1992; Stein and Lian, 1993; Hayatullina et al., 2018).

**FIGURE 1:**
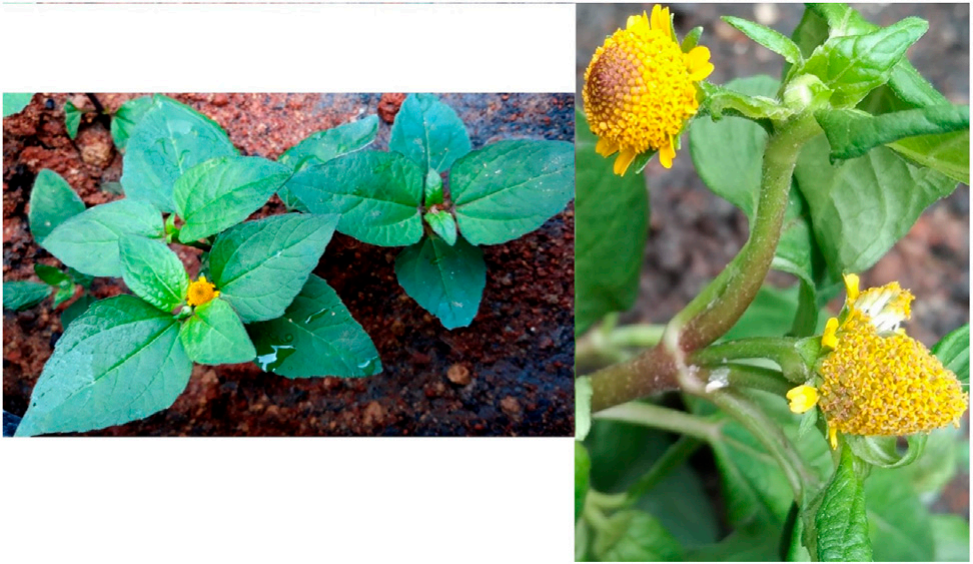
Image of *Blainvillea acmella* (L.) Philipson [Asteraceae] plant with yellow flowers.

*Blainvillea acmella* (L.) Philipson (*B. acmella*) [Asteraceae], synonym as *Spilanthes acmella* (L.) L. (Figure 1) is a herbal plant belonging to the Asteraceae family (Abdul Rahim et al., 2021). The flowers and leaves of *B. acmella* have a pungent taste and cause tingling and numbness on the tongue. Other than being used as a spice for appetizers, it is also traditionally used to treat toothache, stomatitis (Nakatani and Nagashima, 1992), wound healing, snakebite remedy (Ong and Nordiana, 1999), and sore throat (Dubey et al., 2013). *B. acmella* plant was also reported to possess anti-malaria activities (Spelman et al., 2011). The extracts have also been used as nutritional supplements and cosmetics to reduce skin wrinkles *via* constriction of blood vessels (Sharma et al., 2011). In Malaysia, *B. acmella* is popularly known as “Subang Nenek or Pokok Getang” and it is consumed as a herbal treatment for tooth pain (Ong and Nordiana, 1999).

Previous studies reported that *B. acmella* extract exhibited antioxidant, antimicrobial, vasorelaxant (Prachayasittikul et al., 2009; Sharma and Arumugam, 2021), anti-inflammatory (Kim et al., 2018), and aphrodisiac (Sharma et al., 2011) activities. In terms of bone activities, it was able to raise alkaline phosphatase levels (Widyowati, 2011). These properties were contributed by the presence of phytochemicals in *B. acmella* extract. Phenolics, coumarin, triterpenoid, stigmasterol, and stigmasteryl glucoside contributed to the antioxidant activity, whereas vasorelaxant properties were enhanced by pentacyclic and 3-acetylaleuritolic acid (Wongsawatkul et al., 2008; Prachayasittikul et al., 2009). Spilanthol and other alkyl amides present in *B. acmella* extract also contributed significantly to its anti-inflammatory (Sharma et al., 2011; Bakondi et al., 2019) and antioxidant (Abeysiri et al., 2013; Sharma and Arumugam, 2021) activities. A recent study also reported that components in *B. acmella* extract such as 1,3-butanediol 3-pyroglutamate, 2-deoxy-d-ribono-1,4-lactone, methyl pyroglutamate, ampelopsisionoside, icariside B1, bezyl-α-1-arabinopyranosyl-(1,6)-β-d-glucopyranoside (Widyowati et al., 2020a), methyl threonolactones, and pyroglutamates contributed to its ALP activity and mineralization of osteoblast MC3T3-E1 cells (Widyowati et al., 2020b).

However, limited studies have been carried out on the effects of *B. acmella* extract on bone cells. Studies have shown that n-butanol and water fractions from ethanol extract (70%) of *B. acmella* leaves increased ALP activity in osteoblast cells *in-vitro* (Widyowati, 2011). In addition, the use of *B. acmella* extracts followed by exercise activity on mice increased the bone formation rate of mouse femur trabecular (Laswati et al., 2015). Methyl threonolactone glucopyranoside group, methyl threonolactonefructofuranoside, methyl pyroglutamates, and amine cell derivatives isolated from *B. acmella* methanol extracts were recently demonstrated to significantly increase the ALP and mineralization activities of osteoblast cells (Widyowati et al., 2020a; Widyowati et al., 2020b).

Osteoporosis is related to the phytochemicals and antioxidant activity in plants. Previous studies have shown that phenolic compounds are the major secondary metabolites in the plant (Swallah et al., 2020) that contribute to antioxidant activity lower the risk of osteoporosis. Flavonoid compounds are the major compounds in the phenolic group with antioxidant activity (Đudarić et al., 2015).

Nonetheless, no study has been conducted to determine the anabolic effects of the ethanol and aqueous extracts of *B. acmella* on osteoblast cells, as well as compounds that may contribute to the antioxidant and bone anabolic activities. In this study, the ethanol and aqueous extracts of *B. acmella* leaves were analyzed phytochemically for phenolic and flavonoid contents, and their antioxidant activities were measured using DPPH, ABTS, and FRAP assays. Purified water and ethanol were chosen as the maceration technique to extract the biological active compound in *B. acmella* leaves. The relationship between phenolic compounds, antioxidant and anabolic activities were determined. The bone anabolic effects of the *B. acmella* extracts on osteoblast cells (MC3T3-E1) were evaluated by measuring osteoblast proliferation, collagen formation, ALP and mineralization activities. Finally, the possible compounds that contribute to both activities were identified using the GCMS and LCTOFMS systems.

The results from this study will provide the data on the anabolic effect of *B. acmella* extracts on the osteoblast cells, MC3T3-E1, and the correlation between phytochemical presence, antioxidant and bone anabolic activities. Compounds identified will contribute to the antioxidant and anabolic activities of *B. acmella*.

## Materials and Methods

The study was approved by the UKM ethical committee (Approval no: UKM PPI/111/8/JEP-2019-293).

### Plant Identification

The plant *Blainvillea acmella* (L.) Philipson [Asteraceae] was collected from Pagoh, Muar, Johor (coordinates 2.136742; 102.765206) of South Malaysia. Botanical identification of *Blainvillea acmella* (L.) Philipson [Asteraceae] plant was conducted by the Herbarium Unit, School of Biological Sciences, Universiti Sains Malaysia (USM). The voucher specimen: No. 11771 was identified by Dr Rahmad Zakaria and Dr. Farah Alia Nordin and deposited at the Herbarium Unit, School of Biological Sciences, USM.

### Moisture Content Determination

The moisture content of *B. acmella* leaves was determined according to the previous method (Manimaran et al., 2019). *B. acmella* leaves were dried at 40°C in the dryer until the weight became constant. The moisture content of the leaves was determined based on the formulation given:

Moisture content of B. acmella(%)=(Original sample weight−Final sample weight)÷(Original sample weight)×100

### Preparation of *B. acmella* Leaves Ethanol and Aqueous Extracts

The *B. acmella* leaves extracts were prepared based on the modified methods (Gavamukulya et al., 2014; Abdul Rahim et al., 2018). Running water was used to wash the whole plant and it was separated into stems, flowers, and leaves. *B. acmella* leaves were consistently dried in the dryer at 40°C and the dried *B. acmella* leaves were grinded into a fine powder with the use of a herb Grinder. For ethanol extract, fine powdered *B. acmella* leaves were macerated in ethanol 95% (100%) while for aqueous extract, *B. acmella* leaves were macerated in purified water (100%). The *B. acmella* plant was extracted at 1 g: 10 ml (w/v) and placed in a 120 rpm shaker at room temperature for three days. Supernatants were collected daily for three days, centrifuged, filtered, lyophilized, and kept in an airtight container at −20°C for further analysis.

### Yield Determination of *B. acmella* Leaves Extracts

The yield of *B. acmella* leaves extracts was calculated by comparing the weight of the lyophilized dried *B. acmella* extract with the weight of the original dried *B. acmella* as in the formulation given below (Nipornram et al., 2018):

The yield of B. acmella  extracts (%)=Weight of lyophilized extract÷ Weight of original dried B. acmella  ×100

### Determination of Polyphenol Content in Ethanol (*BA*E) and Aqueous (*Ba*A) Extracts of *B. acmella* Leaves

#### Total Phenolic Content (TPC) of *Ba*E and *Ba*A

The TPC of the extracts was determined using Folin-Ciocalteu (FC) method with modification (Luo et al., 2018). *Ba*E and *Ba*A (2 mg/ml) were dissolved in purified ethanol 95% and purified water, respectively and then filtered using a 0.2 μm filter (Polyethersulfone, FisherbrandTM). The extracts (20 µl) were then mixed with Folin-Ciocalteau reagent (80 µl), homogenized, and incubated at room temperature for 4 min. Disodium carbonate (Na_2_CO_3_) solution (100 μl, 7.5% w/v) was then added, followed by incubation at room temperature for 1 h, and the absorbance was measured at 750 nm using a microplate reader (Biotek, San Diego, United States). A standard solution of gallic acid was used for calibration curve preparation (0.3, 0.6, 1.2, 2.3, 4.7, 9.4, 18.8, 37.5, 75, 150, and 300 mg/l). The total phenolic content was expressed as mg gallic acid equivalents (mg GAE/g) of dry weight.

#### Total Flavonoid Content (TFC) of *Ba*E and *Ba*A

The TFC of *B. acmella* leaves extracts was determined using the aluminum chloride colorimetric method with modification from previous studies (Chandra et al., 2014; Luo et al., 2018). *Ba*E and *Ba*A (2 mg/ml) were dissolved in purified ethanol 95% and purified water, respectively, followed by filtration using a 0.2 μm filter (Polyethersulfone, FisherbrandTM). The extracts (50 µl) were then mixed with distilled water (220 µl) and aluminum chloride (AlCl_3_) solution (15 μl, 10% w/v), followed by incubation at room temperature for 15 min, and the absorbance was measured at 405 nm using a microplate reader (Biotek, San Diego, United States). The flavonoid content of each extract was calculated using a standard calibration of quercetin (0.2, 0.4, 0.8, 1.6, 3.1, 6.3, 12.5, 25, and 50 mg/l), whereas the total flavonoid content was expressed as mg quercetin equivalent (mg QE/g) of dry weight.

### Antioxidant Activity of *Ba*E and *Ba*A

#### 2,2-Diphenyl-1-Picrylhydrazyl Radical Scavenging Assay

Antioxidant activity of extracts was measured using DPPH radical scavenging assay in 96-well plates with modified methods from previous studies (Barrientos et al., 2020). *B. acmella* leaves extract (0.1, 0.2, 0.3, 0.7, 1.3, 2.6, 5.3, 10.5, 21.1, 42.1 and 84.2 mg/l) were mixed with ethanolic DPPH and incubated for 30 min at room temperature. L-ascorbic acid was used as a positive control. The absorbance was measured at 517 nm using a microplate reader (Biotek, San Diego, United States). The result was expressed as the percentage of DPPH radical inhibition at 50% (IC_50_). Antioxidant activity was calculated based on the formulation given:

DPPH radical scavenging activity (%) =(Absorbance of the control−Absorbance of the sample)÷(Absorbance of the control)×100 

#### 2,2′-Azino-Bis-(3-Ethylbenzothiazoline-6-sulfonic acid) (ABTS) Radical Scavenging Assay

ABTS assay of the extracts was measured with modification as described by previous authors (Paranagama et al., 2020). The ABTS radical cation (ABTS +) was produced by reacting a 7 mM ABTS (5 ml) stock solution with 140 mM potassium persulfate (88 µl) and the mixture was incubated at room temperature in the dark for 16 h. The ABTS + solution was then diluted with ethanol to obtain an absorbance of 0.70 at 734 nm. The assay was carried out by mixing *B. acmella* extracts (40 μl) with ABTS + solution (100 μl) and incubated at room temperature for 6 min. L-ascorbic acid was used as a positive control. The result was expressed as the percentage of ABTS radical inhibition at 50% (IC_50_). ABTS radical scavenging activity was calculated based on the formulation given:

ABTS radical scavenging activity (%)=(Absorbance of the control−Absorbance of the sample)÷(Absorbance of the control)×100 

#### Ferric Reducing Antioxidant Power Assay

FRAP assay of the extracts was measured with modification as described by previous researchers (Paranagama et al., 2020). The FRAP reagent was freshly prepared by mixing TPTZ (2,4,6-tri (2-pyridyl)-1,3,5-triazine) solution (10 mM) in hydrochloric acid (HCL) (40 mM), ferric chloride (FeCl_3_) (20 mM), and acetate buffer (300 mM) (pH 3.6) at ratios of 1:1:10 (v/v/v). The extracts (40 μl) were then mixed with FRAP reagents (150 µl), incubated at room temperature for 15 min, and the absorbance was measured at 593 nm. L-ascorbic acid solutions (0.005, 0.01, 0.02, 0.04, 0.1, 0.1, 0.2, 0.3, 0.6, 1.2, 2.4, 4.9, 9.8, 19.5 and 39.1 mg/l) were used as the standard curve and expressed as mg L- ascorbic acid equivalents/g of dry weight extracts (mg AAE/g).

### Effects of *Ba*E and *Ba*A on MC3T3-E1 Cells Proliferation and Differentiation

#### Cell Culture Conditions

Mouse osteoblast cells (MC3T3-E1) (ATCC, Rockville, MD, UDA) were grown in alpha Modified Eagle Medium (α-MEM) supplemented with 10% (v/v) fetal bovine serum (FBS) and 1% (v/v) antibiotic-antimycotic (Gibco Life Technologies, Ins., Grand Island, NY, United States). The cells were grown in a 75 cm^2^ flask in a humidified atmosphere of 95% air with 5% CO_2_ at 37°C and the growth medium was changed every 3 days. Once the cells reached 90% confluence, the cells were detached using 0.25% trypsin-EDTA. The cell counting procedure was performed using a hemocytometer (Hirschmann techcolor, Eberstadt, Germany) and viewed under an inverted microscope (CKX41, Olympus, Selangor, Malaysia).

#### Cytotoxicity Analysis and Effects of *B. acmella* Extracts on Cell Proliferation

MTT assay was used to assess cell metabolic activity according to the protocol kit (G4100, Promega). Briefly, the cells were seeded in 96-well plates (1 × 10^3^ cells/cm^2^) and treated with *B. acmella* extracts (1500, 750, 375, 187.5, 93.75, 46.88, 23.44, 11.72, 5.86 and 2.93 µg/ml) at 37°C with 5% CO_2_. At designated time points (1, 3, 5, and 7 days), dye solution (10 µl) was added to the cell culture, followed by 3 h of incubation period in the incubator at 37°C to allow cellular tetrazolium conversion. Then, the cell-culture media was mixed with a stop solution. The absorbance was measured with a microplate reader (Biotek, San Diego, United States) at a wavelength of 570 nm. For each incubation period, the percentage of cell proliferation was calculated based on the formulation given:

Cell proliferation (%) =(Absorbance of treated cells−Absorbance of the blank) ÷(Absorbance of the control−Absorbance of the blank)×100 

#### Sirius Red Assay

Collagen content was determined using the Sirius red-based colorimetric assay based on a method modified from previous studies (Ahmad et al., 2018). MC3T3-E1 cells were seeded on a 24-well plate (1 × 10^5^ cells/cm^2^) and incubated at 37°C with 5% CO_2_. The cells were treated at 80% confluence in the presence of osteogenic media and incubated with various concentrations of *Ba*E (2.92 and 23.44 µg/ml), and *Ba*A (5.85 and 11.72 µg/ml) for 7 and 14 days. The treatment media was changed every 3 days. After the incubation periods, the cell layer was washed with Dulbecco’s phosphate-buffered saline (DPBS) and air-dried overnight in an incubator. The cells were then stained with Sirius red dye reagent (0.1% Sirius red in picric acid) for 1.5 h in the dark under mild shaking. Stained cells were washed with hydrochloric acid (HCL) (0.01 M) to remove all non-bounded dye until the solution becomes colorless. The stain cells were dissolved in a sodium hydroxide (NaOH) (0.01 M) for 30 min with mild shaking. The absorbance was measured at 540 nm using a multiskan spectrum (Thermo Scientific, Corston, Bath, United Kingdom). The percentage of collagen was calculated based on the formula given:

Percentage of collagen (%)=  Absorbance of treated cells ÷ Absorbance of untreated (control) cells×100 

#### Alkaline Phosphatase (ALP) Activity

ALP activity was analyzed with the use of an Alkaline Phosphatase Assay Kit (ab83369, Abcam Inc., Cambridge, United Kingdom). Mouse osteoblast cell (MC3T3-E1) was seeded on a 24-well plate (1 × 10^5^ cells/cm^2^) and incubated at 37°C with 5% CO_2_. The cells were treated at 80% confluence in the presence of osteogenic media and incubated with various concentrations of *Ba*E (2.92 and 23.44 µg/ml), and *Ba*A (5.85 and 11.72 µg/ml) for 7 and 14 days. The media was changed every 3 days. After the incubation periods, the cell layer was washed with cold DPBS and resuspended in ALP buffer (50 µL). The cells were then homogenized using an ultrasonic homogenizer (150/VT, Virginia, United States) for 2–3 s and kept on ice. Next, the cells were centrifuged at 4°C, 12,000 rpm for 15 min. The supernatants were collected for ALP activity and measured at 405 nm using a multiskan spectrum (Thermo Scientific, Corston, Bath, United Kingdom). The results were expressed as ALP activity of treated cells to untreated (control) cells:

$$\mathrm{ALP}\;\mathrm{activity}\;\left(\frac{\mathrm{nmol}}{\mathrm{well}}\mathrm{pNPP}\right)=\mathrm{Absorbance}\;\mathrm{of}\;\mathrm{treated}\;\mathrm{cells}\;÷\mathrm{Absorbance}\;\mathrm{of}\;\mathrm{untreated}\;\left(\mathrm{control}\right)\;\mathrm{cells}\times 100$$

#### Alizarin Red S Assay

Calcified calcium nodule formation was determined using Alizarin Red S staining based on a method modified from Ahmad et al. (2018). MC3T3-E1 cells were seeded on a 24-well plate (1 × 10^5^ cells/cm^2^) and incubated at 37°C with 5% CO_2_. The cells were treated at 80% confluence in the presence of osteogenic media and incubated with various concentrations of *Ba*E (2.92 and 23.44 µg/ml) and *Ba*A (5.85 and 11.72 µg/ml) for 14 and 21 days. The treatment media was changed every 3 days. After the incubation periods, the cell layer was washed with DPBS and then fixed with paraformaldehyde (4%) for 15 min at room temperature. The cells were then washed with DPBS and stained with Alizarin Red S solution 40 mM (pH 4.2) for 30 min at room temperature. Stained cells were washed with water to remove all non-bounded dye until the solution becomes colorless. The stained cells were dissolved in cetyl peridium chloride (10%) in sodium dihydrogen phosphate (10 mM) for 30 min with mild shaking. A multiskan spectrum (Thermo Scientific, Corston, Bath, United Kingdom) was used to measure the absorbance at 570 nm. The percentage of mineralization was calculated based on the formula given:

Percentage of mineralization (%) =Absorbance of treated cells ÷Absorbance of untreated (control) cells×100 

### Identification of Compound in *Ba*E using Gas Chromatography Mass Spectrometry (GCMS) and Liquid Chromatography Time of Flight Mass Spectrometry (LCTOFMS) System

#### Sample Preparation for *Ba*E

*Ba*E was dissolved in ethanol (95%) at a concentration of 10 mg/ml and 25 mg/ml and filtered using Whatman PVDF Syringe filter, 0.2 µm (Polyethersulfone, FisherbrandTM). The extracts were then transferred into vials for GCMS and LCTOFMS analysis respectively.

#### Instrumentation and Chromatographic Condition of GCMS

Chemical compound identification was performed with some modifications (Anholeto et al., 2017). GCMS system consisting of an Agilent 6890 gas chromatography coupled with an Agilent 5973 mass spectrometer. Separation of compounds was carried out using HP-5 MS capillary column (30 m × 0.25 mm × 0.25 μm). The analysis was set at: injector at 220°C; split mode at 1:10 ratio; oven temperature at 40°C–250°C (2.5°C min^−1^); carrier gas: helium at a flow rate of 1.0 ml min^−1^; injection volume at 1 µl. MS condition was set with an ionizing voltage at 71 eV. The ionization source was set at 250°C. The scanning range was set between 30 amu for low mass and 1,000 amu for high mass with a solvent delay of 5.00 min. The total ion chromatograms (TICs) and mass spectra were recorded using MSD Chemstation Data Analysis software. Mass spectral identification of unknown chemical compounds in extracts was performed by comparing with NIST library with match quality above 85% (Samsurrijal et al., 2019).

#### Instrumentation and Chromatographic Condition of LCTOFMS

Phenolic compounds were determined using the liquid chromatography time of flight mass spectrometry (LCTOFMS) system. The detector used was a mass spectrometer with an ESI interface. Separation was carried out by injecting 10 µL aliquots into a C18 reversed-phase column (Zorbax, 150 mm, 4.6 µm) at room temperature. The elution was performed as reported in Table 1. The mass spectrometer operated in full-scan MS mode, from m/z 100 to 1,200 in positive modes. The electrospray interface (ESI) provided nebulization by applying 3.0 kV ion spray voltage and capillary temperature of 200°C. Instrument control, data acquisition was provided by Masslynk 4.1 software. Data processing and chemical compound identification in extracts were performed by MS-DIAL software (RIKEN, version 4.7) (http://prime.psc.riken.jp/compms/msdial/main) with a score above 80%, peak detection at 5,000 amplitude, and MS identification using Vaniya Fiehn Natural Product Library.

**TABLE 1:** Chromatographic gradient for LCTOFMS analysis.

Time (minutes)	A (Acetonitrile)	B (1% Formic acid in water)
0	10	90
15	11	89
18	15	85
20	20	80
23	30	70
25	35	65
28	40	60
30	45	55
50	60	40
60	10	90

### Statistical Analysis

Results were presented as mean ± standard deviation (SD) and mean ± standard error of the mean (SEM). The slope of the calibration curve and the coefficient of determination (*R* ^2^) were obtained using MS Excel version 2110. Data obtained for cytotoxicity, proliferative, and antioxidant activity were normally distributed using Shapiro-Wilk and statistically processed with a one-way analysis of variance (ANOVA) test with Tukey HSD post-hoc tests to determine the significant differences among samples. (*p* < 0.05) was accepted as statistically significant. Control (untreated) and treatments groups were compared for cytotoxicity and proliferative activities. Statistical relationships with a linear model between TPC, TFC, DPPH, FRAP, ABTS, proliferation, ALP, collagen and mineralization were carried out using Pearson’s correlation analysis. This analysis was performed to assess if two different variables are associated. All statistical analyses were conducted using the Statistical Package for Social Science (IBM SPSS statistical software version 26, IBM, New York, NY, United States).

## Results

### Percentage of Moisture Content and Extraction Yield of Ethanol and Aqueous Extract of *B. acmella* Leaves

The residual moisture content in *B. acmella* leaves was 84.56% ± 1.45. Extraction yield of *Ba*E was 7.04% ± 1.30 and *Ba*A was 11.97% ± 0.31.

### Total Phenolic Content (TPC) and Total Flavonoid Content (TFC)

TPC and TFC of *Ba*E were 30.25 ± 0.85 mg GAE/g dry weight and 30.06 ± 1.18 mg QE/g dry weight, respectively. On the other hand, the TPC and TFC of *Ba*A were 24.38 ± 2.15 mg GAE/g dry weight and 2.41 ± 0.12 mg QE/g dry weight, respectively. Table 2 shows the results of TPC and TFC of *B. acmella* leaves extracts. TPC and TFC of *Ba*E were significantly higher (*p* < 0.01) than *Ba*A.

**TABLE 2:** TPC and TFC of *B. acmella* extracts.

Extracts	TPC (mg GAE/g dry weight)	TFC (mg QE/g dry weight)
*Ba*E	30.25 ± 0.85^a^	30.06 ± 1.18^a^
*Ba*A	24.38 ± 2.15^b^	2.41 ± 0.12^b^
Calibration	Y = 0.004x + 0.001 (*R* ^2^ = 0.999)	Y = 0.035x + 0.032 (*R* ^2^ = 0.999)

Ba*E*: B. acmella ethanol extract; Ba*A*: B. acmella aqueous extract. Values represent mean ± standard deviation, *n* = 6. Different superscript letters show significant differences (*p* < 0.05) in the same analysis. Calibration curves: Y = mx+c; Y = absorbance, x = concentration of gallic acid (GAE) or quercetin (QE).

### Antioxidant Activity (DPPH, ABTS and FRAP Assays)

Antioxidant activity for DPPH and ABTS assays in *B. acmella* extracts was reported as the amount of antioxidants required to decrease the initial concentration at 50% (IC_50_). Table 3 shows the IC_50_ value of *B. acmella* extracts in DPPH and ABTS assays. DPPH assay revealed that *Ba*E contributed to significantly lower IC_50_ values than *Ba*A. Meanwhile, for the ABTS assay, no significant differences (*p* > 0.05) in the IC_50_ values were observed for *Ba*A and *Ba*E. For FRAP assay, *Ba*E contributed to significantly higher antioxidant activity (*p* < 0.01) than *Ba*A.

**TABLE 3:** DPPH, ABTS and FRAP activities of *Ba*E and *Ba*A.

Value	Samples/Antioxidant assays	*Ba*A	*Ba*E	L- ascorbic acid
Antioxidant activity, IC_50_ (µg/ml)	DPPH	860.67^a^	476.71^b^	20.25^c^
	ABTS	192.56^a^	201.49^a^	13.70^b^
Antioxidant activity (mg AAE/g)	FRAP	3.31 ± 0.53^a^	56.01 ± 6.46^b^	-

Ba*E*: B. acmella leaves ethanol extract; Ba*A*: B. acmella leaves aqueous extract. mg AAE/g: mg L-ascorbic acid equivalent/g. Value represents mean ± standard deviation, *n* = 6; Different superscript letters show significant differences between means in the same assays at *p* < 0.05.

### Cytotoxicity Analysis of *B. acmella* Extracts on Osteoblast Cells (MC3T3-E1)

**FIGURE 2:**
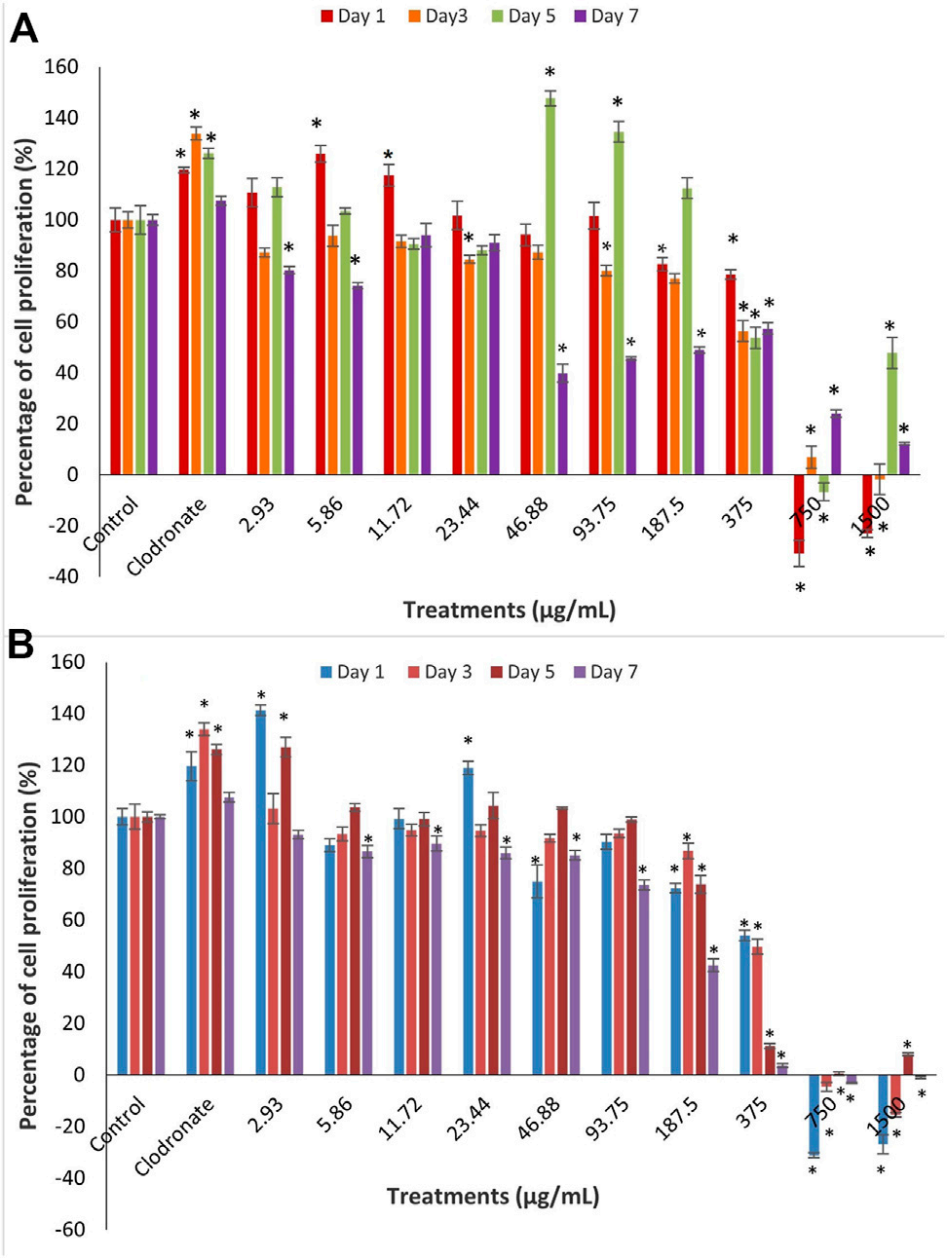
Cell proliferation of MC3T3-E1 (osteoblast) cells after treatment with different concentrations of *B. acmella* **(A)** aqueous (*Ba*A) and **(B)** ethanol (*Ba*E) extracts at Day 1, Day 3, Day 5 and Day 7 of incubation. Results (optical densities) were calculated as the percentage of unexposed control cultures and represented by mean ± SEM. *p-value less than 0.05 (*p* < 0.05) indicated significant difference when compared to untreated cells (control) (*n* = 6) at same incubation times.

The cytotoxicity analysis of MC3T3-E1 cells after treatment with *Ba*A (A) and *Ba*E (B) leaves, at concentrations of 2.93 µg/ml to 1,500 µg/ml at day 1, 3, 5, and 7 are shown in Figure 2.

Results showed that concentrations of extracts at 750 µg/ml to 1,500 µg/ml for all days and extracts at 46.88, 93.75 and 187.5 µg/ml on day 7 were toxic to cells, with less than 50% of viable cells compared to control for all days. Meanwhile, no cytotoxicity was found for both extracts at concentrations of 2.93 µg/ml to 187.5 µg/ml with more than 50% of viable cells at all days except at 46.88, 93.75 and 187.5 µg/ml on day 7. There were fluctuations in the percentage of cell proliferation from low to high doses at all treatment days. On day 1, *Ba*E induced a significantly higher cell proliferation than control (*p* < 0.05) at the treatment concentrations of 2.93 µg/ml (141.32%) and 23.44 µg/ml (118.99%). On day 5, *Ba*A caused a significantly higher cell proliferation than the control (*p* < 0.05) at treatment concentrations of 46.88 µg/ml (147.76%) and 93.75 µg/ml, which were about 134.53%, respectively. When compared, *Ba*E induced higher cell proliferation at lower concentrations and earlier days than *Ba*A.

Based on these findings, *Ba*E concentrations (2.93 µg/ml and 23.44 µg/ml) and *Ba*A concentrations (5.8 µg/ml and 11.72 µg/ml), which caused the highest significant cell proliferation on day 1 were chosen for further assessments on the differentiation of MC3T3-E1 cells.

### Effects of *B. acmella* Extracts on Cell Proliferation Activity of Osteoblast Cells (MC3T3-E1)

Clodronate is the earliest group of non-nitrogen bisphosphonates that have been widely used in the clinical treatment for bone-related diseases by enhancing bone inhibition (Koch et al., 2011; Frediani and Bertoldi, 2015; Nardi et al., 2016; Pherwani and Sathaye, 2020). Bisphosphonates reacted with osteoblast cells and altered bone metabolism by stimulating bone formation (Corrado et al., 2017). Previous studies reported that clodronate affects the anabolic activity of bone cells by enhancing the differentiation and mineralization of MC3T3-E1 cells (Itoh et al., 2003; Koch et al., 2011; Maruotti et al., 2012). Therefore, clodronate has been used as a positive control to study the effects of *B. acmella* extracts on MC3T3-E1 cells.

**FIGURE 3:**
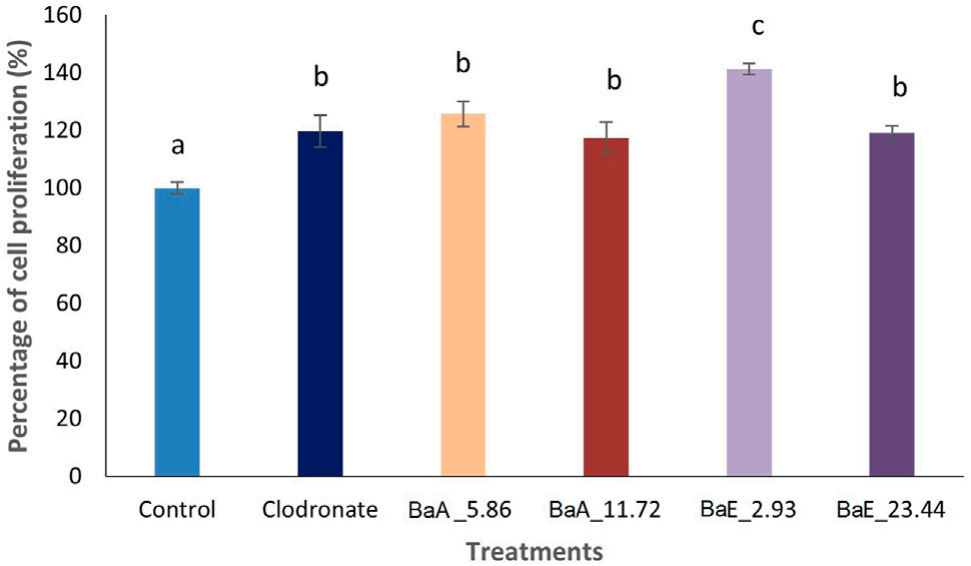
Cell proliferation activity of MC3T3-E1 (osteoblast) cells after treatment with different concentrations of *B. acmella* aqueous (*Ba*A) and ethanol (*Ba*E) extracts at Day 1. BaE_2.93 (*Ba*E at 2.93 µg/ml), BaE_23.44 (*Ba*E at 23.44 µg/ml), BaA_5.86 (*Ba*A at 5.86 µg/ml) and BaA_11.72 (*Ba*A at 11.72 µg/ml). Results (optical densities) were calculated as mean ± SEM. Different alphabets indicated significance differences (*p* ≤ 0.05) (*n* = 6) between treatments.

Results in Figure 3 showed the cell proliferation activity of MC3T3-E1 cells after treatment at the determined concentration of *B. acmella* extracts on day 1. The highest cell proliferation was significantly observed at the *Ba*E concentration of 2.93 µg/ml, which was about 141.32% compared to clodronate and other treatment concentrations. As for *Ba*A, significantly (*p* < 0.05) higher cell proliferation than control was observed at the concentrations of 5.86 µg/ml, which was about 125.94%. However, no significant difference (*p* > 0.05) was found between BaA_5.86, BaA_11.72 and BaE_23.44.

**FIGURE 4:**
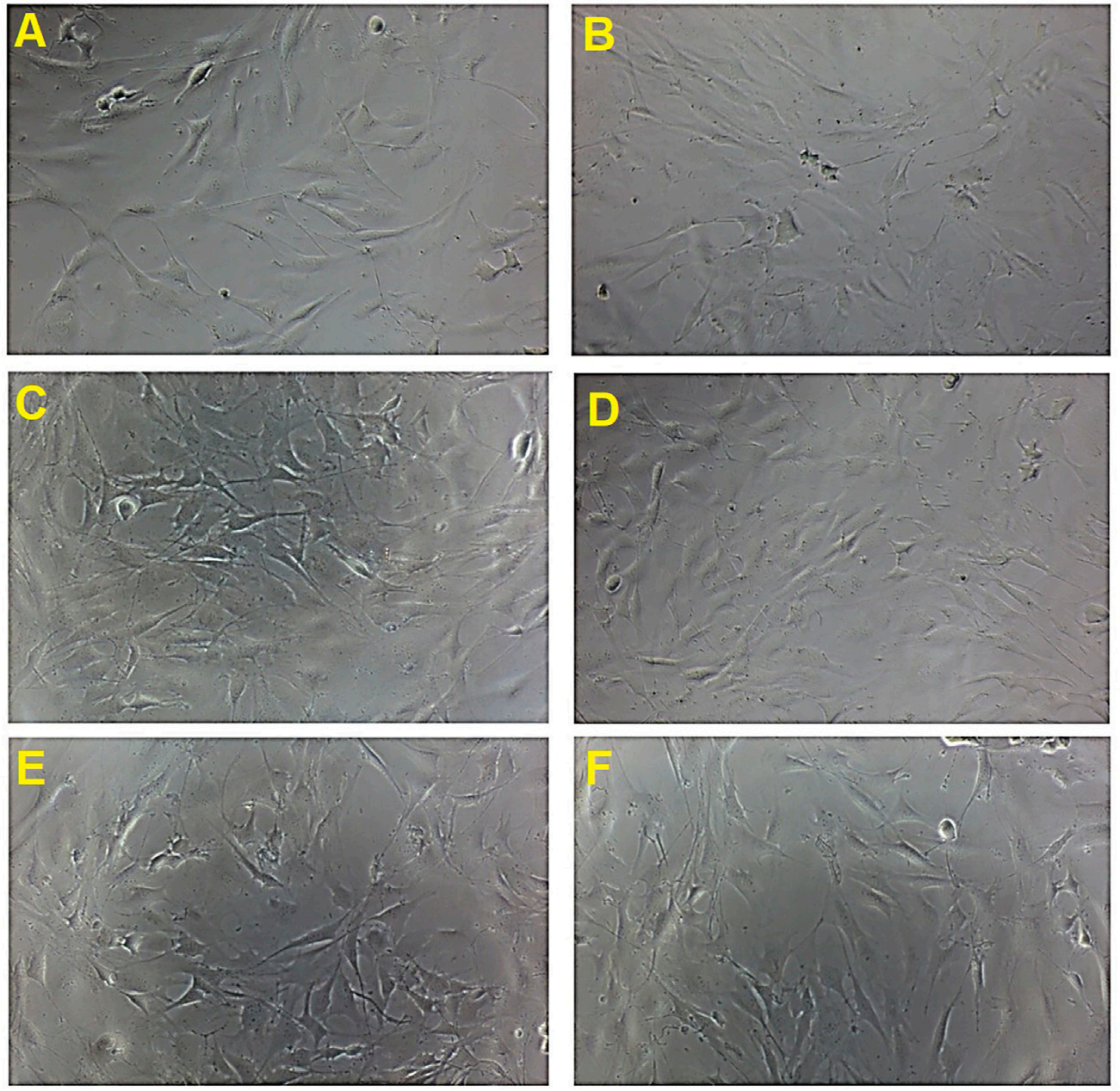
Cell proliferation activity and morphological changes in MC3T3-E1 cells treated *with B. acmella* extracts. **(A)**: Control (untreated cells); **(B)**: Positive control (Clodronate); **(C)**: BaE_2.93 (*Ba*E at 2.93 µg/ml), **(D)**: BaE_23.44 (*Ba*E at 23.44 µg/ml), **(E)**: BaA_5.86 (*Ba*A at 5.86 µg/ml) and **(F)**: BaA_11.72 (*Ba*A at 11.72 µg/ml) at Day 1 compared to control (untreated cells) at 100x magnification.

The morphological features of MC3T3-E1 cells treated with the four selected concentrations of *B. acmella* extracts (BaA_5.86, BaA_11.72, BaE_2.93 and BaE_11.72), on day 1 were compared with controls using an inverted microscope and the micrographs were shown in Figure 4.

The micrographs showed that MC3T3-E1 cells have a flat, polygonal appearance and were uniformly thin with smooth extended cytoplasm. It also has a spindle-shaped morphology with a fibroblastic appearance. The number of cells increased on day 1 after treatment with *B. acmella* extracts compared to control and clodronate. The result obtained was also in agreement with the MTT proliferation assay, as shown in Figure 3. These findings indicated that the osteoprotective effect of *B. acmella* extracts were *via* amplification of osteoblast proliferation. These results also revealed that 2.93 µg/ml was the most compatible concentration of *B. acmella* followed by others *B. acmella* (BaE_23.44, BaA_5.86 and BaA_11.72) to provoke a significant proliferation of MC3T3-E1 cells compared to control (untreated cells).

### Effects of *B. acmella* Extracts on Differentiation Activities of Osteoblast Cells (MC3T3-E1)

#### Collagen Determination and Alkaline Phosphatase Activity

**FIGURE 5:**
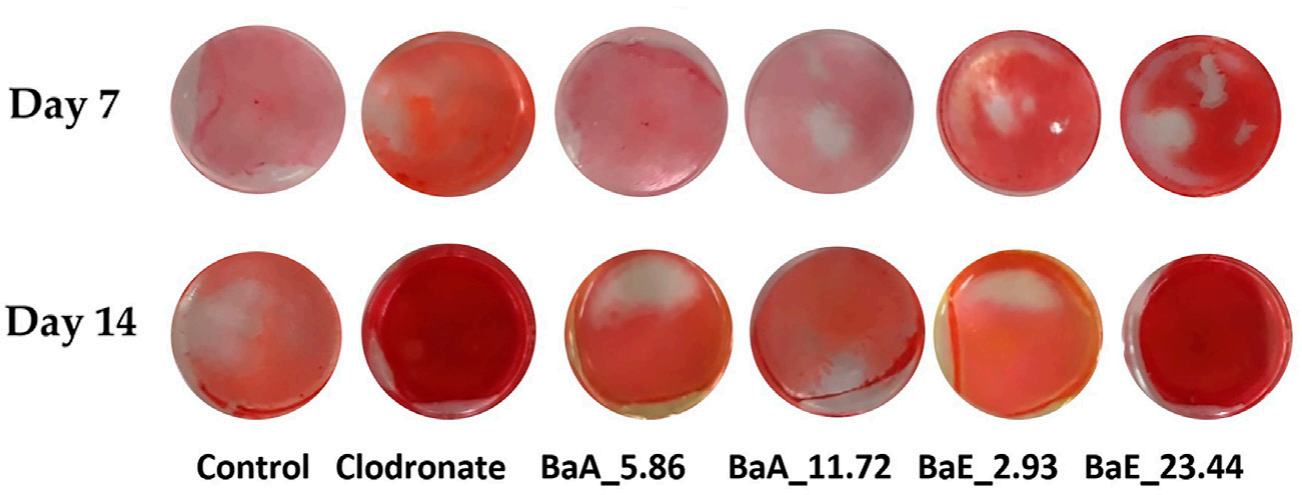
Photomicrographs of collagen-stained cells in *B. acmella* aqueous (*Ba*A) and ethanol (*Ba*E) extracts at Day 7 and Day 14. Control (untreated cells), Positive control (Clodronate), BaE_2.93 (BaE at 2.93 μg/ml), BaE_23.44 (BaE at 23.44 μg/ml), BaA_5.86 (BaA at 5.86 μg/ml) and BaA_11.72 (BaA at 11.72 μg/ml).

The effects of *Ba*E and *Ba*A on early osteoblast differentiation were first assessed by measuring the percentage of collagen content and ALP activity of MC3T3-E1 cells at 7 and 14 days of treatment. The percentage of collagen secreted by osteoblast was estimated by Sirius red staining with red color formation by the reaction of sulfonic acid in Sirius red and the collagen fibers. Figure 5 shows the photomicrographs of MC3T3-E1 cells with or without treatment with *B. acmella* leaves extracts and stained with Sirius red.

The red color staining of MC3T3-E1 cells receiving *B. acmella* leaves extracts treatment was more intense and widely distributed in the whole plate region on day 14 compared to the control cells. *Ba*E at a concentration of 23.44 µg/ml showed the highest red color intensity due to collagen depositions. The stained cells were further dissolved, and the absorbance of *B. acmella* leaves extracts was measured. Furthermore, the percentage of collagen content was calculated by comparing it with the untreated cells (control).

**FIGURE 6:**
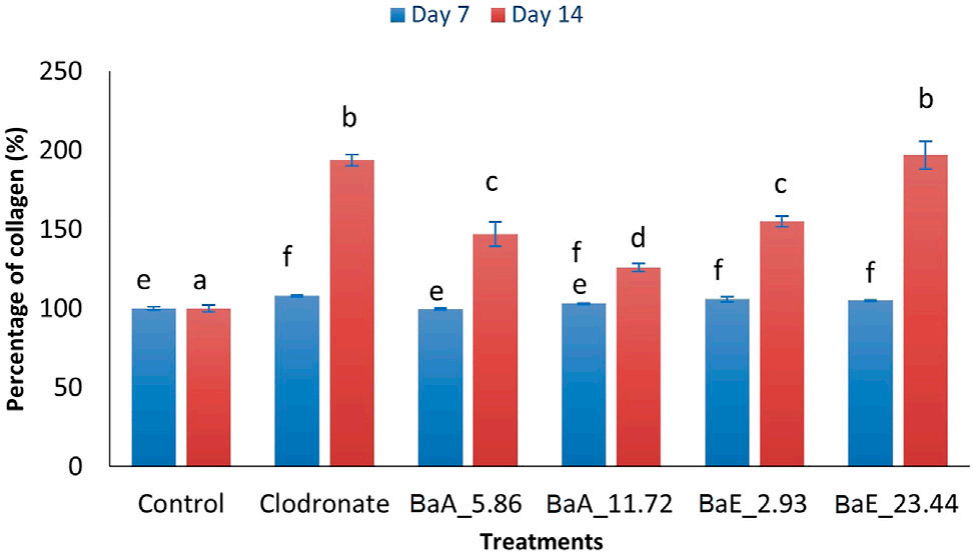
Percentage of collagen in *B. acmella* aqueous (*Ba*A) and ethanol (*Ba*E) extracts. Control (untreated cells), Positive control (Clodronate), BaE_2.93 (*Ba*E at 2.93 µg/ml), BaE_23.44 (*Ba*E at 23.44 µg/ml), BaA_5.86 (*Ba*A at 5.86 µg/ml) and BaA_11.72 (*Ba*A at 11.72 µg/ml). Different alphabet shows significant different (*p* < 0.05) between treatments in a same day. The data are presented as mean ± SEM (*n* = 6).

In line with the collagen staining results, *B. acmella* treatments produced an increase in collagen synthesis after 14 days of treatment as shown in Figure 6.

Results showed that the collagen percentage of cells increased significantly (*p* < 0.05) from day 7 to day 14 with clodronate and *B. acmella* treatments compared to control cells. On day 7, the collagen percentage of *Ba*E-treated cells (2.93 and 23.44 µg/ml) were significantly higher (*p* < 0.05) than control cells. However, no significant difference was found between the two concentrations of *Ba*E-treated cells. On day 14, the percentage of collagen was significantly increased from 2.93 µg/ml to 23.44 µg/ml.

With aqueous extract (*Ba*A) treatment, the collagen content was significantly increased (*p* < 0.05) compared to control at concentration of 5.86 µg/ml and 11.72 µg/ml on day 14. Meanwhile, no significant difference was found for both aqueous extracts of *B. acmella* on day 7 compared to the control. The highest collagen content was found on day 14 with *Ba*E at the concentration of 23.44 µg/ml, which was about 196.81% higher than the control. However, no significant difference was observed between *Ba*E at the concentration of 23.44 µg/ml and clodronate. Similar results were found on day 7 as the treatment with ethanol extracts (BaE_2.93 and BaE_23.44) showed higher collagen content compared to aqueous extracts (BaA_5.86 and BaA_11.72), which was not significantly different (*p* > 0.05) compared to clodronate.

**FIGURE 7:**
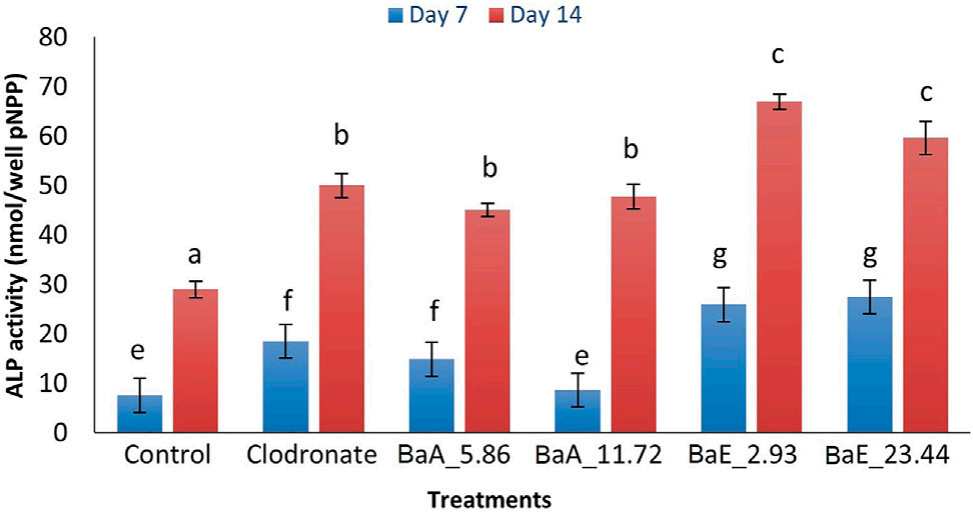
ALP activity in *B. acmella* aqueous (*Ba*A) and ethanol (*Ba*E) extracts. Control (untreated cells), Positive control (Clodronate), BaE_2.93 (*Ba*E at 2.93 µg/ml), BaE_23.44 (*Ba*E at 23.44 µg/ml), BaA_5.86 (*Ba*A at 5.86 µg/ml) and BaA_11.72 (*Ba*A at 11.72 µg/ml). Different alphabets shows significant different (*p* < 0.05) between treatments in a same day. The data are presented as mean ± SEM (*n* = 6).

Figure 7 shows the percentage of ALP activity of *B. acmella* leaves for 7 and 14 days. The ALP activity was increased from day 7 to day 14 for all treatments. Ethanol extracts showed higher ALP activity compared to aqueous extract. All treatment groups had significantly higher ALP activity than control for all days except 11.72 µg/ml of *Ba*A on day 7. The highest ALP activity was found on day 14 with *Ba*E at the concentration of 2.93 µg/ml, which was about 2.31 and 1.35 times more than control and clodronate, respectively. However, no significant difference was found between ethanol extract concentrations of 2.93 µg/ml and 23.44 µg/ml, when the same-day treatment was compared. Likewise, no significant difference was found between aqueous extracts (BaA_5.86 and BaA_11.72) on day 14.

#### Mineralization (Calcium Depositions)

**FIGURE 8:**
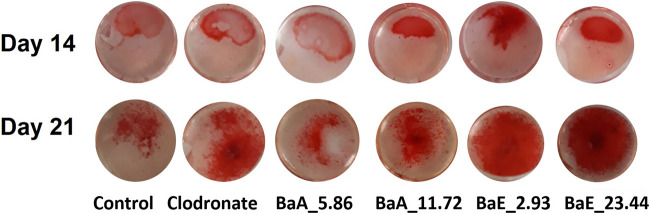
Photomicrographs of mineral depositions stained with Alizarin Red staining in *B. acmella* aqueous (*Ba*A) and ethanol (*Ba*E) extracts on Day 14 and Day 21. Control (untreated cells), Positive control (Clodronate), BaE_2.93 (BaE at 2.93 μg/ml), BaE_23.44 (BaE at 23.44 μg/ml), BaA_5.86 (BaA at 5.86 μg/ml) and BaA_11.72 (BaA at 11.72 μg/ml).

The deposition of calcium noduls in MC3T3-E1 cells after treatment with *Ba*E (2.93 and 23.44 µg/ml) and *Ba*A (5.86 and 11.72 µg/ml) were determined using Alizarin Red staining on day 14 and day 21 as shown in Figure 8.

**FIGURE 9:**
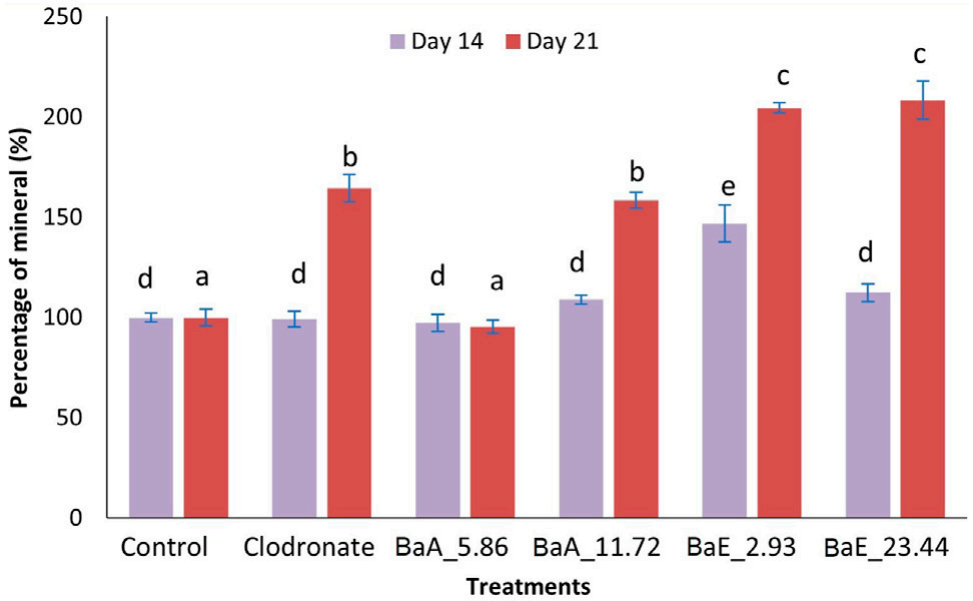
Percentage of mineral in *B. acmella* aqueous (*Ba*A) and ethanol (*Ba*E) extracts. Control (untreated cells), Positive control (Clodronate), BaE_2.93 (*Ba*E 2.93 µg/ml), BaE_23.44 (*Ba*E 23.44 µg/ml), BaA_5.86 (*Ba*A 5.86 µg/ml) and BaA_11.72 (*Ba*A 11.72 µg/ml) different alphabets show significant different (*p* < 0.05) between treatments in a same day. The data are presented as mean ± SEM (*n* = 6).

Alizarin Red S staining revealed the calcium in the bone nodules by forming a bright-colored alizarin red s-complex. It shows evidence of matrix mineralization in the calcified region. No observable differences were noted between the group on day 14 except at BaE_2.93. On day 21, the red staining in MC3T3-E1 cells was intense with 2.93 and 23.44 µg/ml of ethanol extracts, followed by 11.72 µg/ml of aqueous extract and clodronate. Furthermore, the percentage of calcium depositions was determined as shown in Figure 9.

Generally, ethanol extracts contributed to a higher percentage of minerals compared to aqueous extracts. On day 14, only 2.93 µg/ml ethanol extract was found to be significantly higher than untreated cells (control). On day 21, both concentrations of ethanol extracts (2.93 µg/ml and 23.44 µg/ml) showed significantly higher mineral percentages (*p* < 0.05) compared to control and clodronate. No significant difference was found between the two concentration of ethanol extracts on day 21.

### Correlation Analysis Between TPC, TFC, Antioxidant (DPPH, ABTS and FRAP) and Anabolic (Cells Proliferation, Collagen, ALP and Mineral) Activities

Table 4 show the Pearson correlation between TPC, TFC, antioxidant and anabolic activities of *B. acmella* using Pearson’s correlation analysis. No significant correlation was found between ABTS and cell proliferation. A highly significant positive correlation was found between 1) TPC, TFC and FRAP assay 2) TFC, cell proliferation, collagen, ALP activity and mineralization 3) TPC and ALP activity and 4) FRAP, cell proliferation, collagen, ALP activity and mineralization 5) TFC and ABTS 6) ABTS, collagen and mineralization. A highly negative correlation was found between 1) TPC, TFC, DPPH and FRAP assay 2) DPPH assay, cell proliferation, collagen, ALP activity and mineralization. A moderate positive correlation was found between 1) TPC, cell proliferation and collagen activity and 2) ALP activity and ABTS assay. A moderate negative correlation was found between ABTS and DPPH assay.

**TABLE 4:** Pearson correlation between TPC, TFC, antioxidant and anabolic activities.

		TFC	DPPH	ABTS	FRAP	% Cell proliferation	% Collagen	ALP activity	% Mineralization
TPC	R	0.887	−0.869	0.574	0.881	0.622	0.643	0.835	0.834
	p	0.000**	0.000**	0.050*	0.000**	0.031*	0.024*	0.001**	0.001**
TFC	R		−0.995	0.718	0.993	0.724	0.810	0.892	0.853
	p		0.000**	0.009**	0.000**	0.008**	0.001**	0.000**	0.000**
DPPH	R			−0.686	−0.985	−0.725	−0.800	−0.889	−0.835
	p			0.014*	0.000**	0.008**	0.002**	0.000**	0.001**
ABTS	R				0.698	0.380	0.888	0.692	0.724
	p				0.012*	0.223	0.000**	0.013*	0.008**
FRAP	R					0.738	0.816	0.887	0.882
	p					0.006**	0.001**	0.000**	0.000**

TPC*:* total phenolic content; TFC: total flavonoid content; DPPH (2,2-diphenyl-1-picrylhydrazyl); ABTS (2,2’-azino-bis (3- ethylbenzothiazoline-6-sulfonate); FRAP (Ferric reducing antioxidant power); P= *p*-value (significant); R = Pearson’s correlation; *n* = 12. **Correlation is significant at the *p* < 0.01 and *correlation is significant at the *p* < 0.05.

### GCMS and LCTOFMS Analysis

**FIGURE 10:**
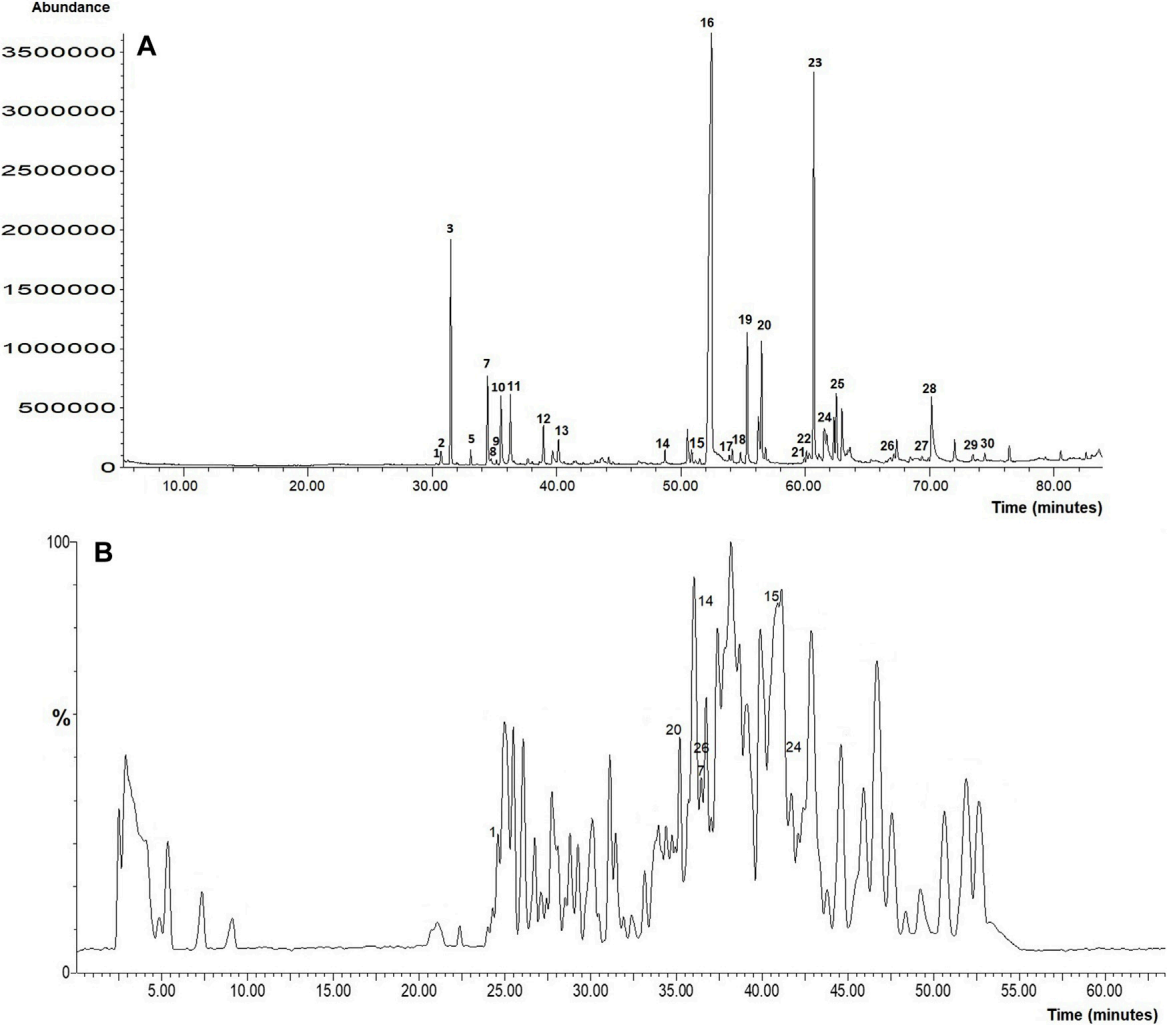
**(A)** GCMS and **(B)** LCTOFMS total ion chromatograms of compounds from *Ba*E. Known compounds were labeled according to Supplementary Table S1 and Supplementary Table S2. Each number labeled at chromatogram referred to the number in Supplementary Table S1 and Supplementary Table S2.

Based on the superior antioxidant and MC3T3-E1 cells proliferation and differentiation activities showed by the ethanol extract of *B. acmella* (*Ba*E), the extract was further analyzed using GCMS and LCTOFMS systems. The identified compounds might be responsible for these actions. Figure 10A and Figure 10B shows the GCMS and LCTOFMS total ion chromatograms (TIC) of compounds from *Ba*E.

Based on the matching quality at 85% (Samsurrijal et al., 2019) and above, GCMS analysis showed that *Ba*E contained 91 chemical compounds, including 30 known and 61 unknown compounds. Alkyl amides (30.93%) was the most abundant group present in *Ba*E extract, followed by terpenoid (16.56%), alkene (4.27%), fatty acid ester (2.95%), phenolic (2.22%), phthalic acid ester (0.69%), and alkana (0.31%) groups. Meanwhile, 19.89% of abundance compounds were contributed by the unknown compounds. Spilanthol (29.53%), the marker compound for *B. acmella* was the major compound in *Ba*E.

Supplementary Table S1 and Supplementary Table S2 shows known biological active compounds in *Ba*E using GCMS and LCTOFMS respectively. Several biological active compounds identified in *Ba*E contribute to the stem cell and osteoblast proliferation, antioxidant and bone anabolic activities. GCMS analysis shows the highest antioxidant activity contributed by alkyl amides (35.09%) from the total abundance of 59.27% antioxidant activity. The highest anabolic activity contributed by diterpenoids (10.98%) from the total abundance (20.57%) of anabolic activity. Diterpenoids (10.98%) contributed to both antioxidant and anabolic activities from the total abundance of terpenoid (17.66%) activity.

LCTOFMS analysis combined with MS-DIAL software at a score of more than 80%, showed that *Ba*E contained 169 chemical compounds, including 32 known and 137 unknown compounds, which includes fatty acid (5.57%), flavonoids (4.95%), alkyl amides (4.00%), amino acids (2.58%), terpenoids (1.95%), guaianes (1.22%), alkaloids (1.18%), vitamin B (1.06%), purine nucleosides (0.57%), methoxy phenols (0.48%), coumarin (0.09%), phenolic acids (0.01%). A total of 5.44% phenolic compounds were detected.

## Discussions

### Moisture Content, Extraction Yield, Phytochemical Content and Antioxidant Activity of *B. acmella* Leaves Extracts

Moisture content and extraction yield are parts of herbs characterization. It is one of the essential quality standards in the standardization process of herbal drugs. The percentage of moisture content of *B. acmella* in this study was 84.56%, higher than *B. acmella* cultivated in India which was 4.32% (Kumar and Ravindra, 2018). A study by Otles and Yalcin (Otles and Yalcin, 2012), showed that the percentage of moisture content in roots, stalks, and stems of netter plants that were planted in various locations ranged from 6.3 to 88.88%. Several factors, such as seasons, geographical origin, and environmental conditions contribute to the moisture content in plants (Azonwade et al., 2018). Therefore, the difference in the percentage of moisture content between the present and previous studies might be due to the variation in seasons, geographical origin, and environmental conditions, such as climate. Tee et al. (2014) reported that the low moisture content of *D. edulis* and *D. rostrata* fruits indicated that the fruits have a high dry matter content and highly nutrient-dense pulp. Thus, these properties might explain the disparities observed between the *B. acmella* leaves investigated in the present study compared to the research by Kumar and Ravindra (2018).

A secondary metabolite is a biologically active compound present in low amounts in plants. Therefore, the extraction method is critical in obtaining a high yield of phytochemicals with minimal changes to the functional properties of the biologically active compounds in the plant. The extraction method employed in this study involved a maceration technique using purified water and ethanol to extract the active compound in *B. acmella* leaves. This method was chosen due to its cost-effectiveness and close resemblance to the traditional extraction method of herbal medicine. Purified water (100%) and 95% ethanol (100%) were used in our extraction method instead of other solvents, such as methanol, hexane, and diethyl ether, as they are green solvents, suitable for human consumption, reusable and nontoxic (Ben Yakoub et al., 2018).

Several factors contributed to the extraction yields, such as the polarity of solvents (Do et al., 2014). The solvent polarity reacts based on the sample properties, analyte chemical properties, and interactions between the matrix and analyte (Dhanani et al., 2017). These factors contribute to the different groups of compounds being extracted (Chandra et al., 2014; Iloki-Assanga et al., 2015; Ghasemzadeh et al., 2018). In this study, water and ethanol were used as solvents for extraction to evaluate the phytochemical and biological activity of *B. acmella*. For standardization, the same cultivated area and batch of *B. acmella* were used*,* and the extraction parameters were fixed during the extraction process.

This was in agreement with the findings by previous study on the successive extraction with petroleum ether, benzene, chloroform, ethanol and water which reported that water extract (3.730%) of *B. acmella* roots, stems and leaves contributed to the highest extraction yield followed by ethanol (2.305%), chloroform (0.825%), petroleum ether (0.736%) and benzene (0.735%) extracts (Tanwer and Vijayvergia, 2010). In a study on a different plant, Do et al. (Do et al., 2014) found that the extraction yield in *Limnophila aromatica* aqueous extract (25.58 ± 1.04%) was higher than ethanol extract (17.03 ± 2.66%)*.* Do et al. (2014) concluded that the extraction yield increased with solvent polarity. The same pattern was found in another plant, *Adansonia digitata* (Ismail et al., 2019). Therefore, the extraction yield of *B. acmella* in the current study increased with solvent polarity, meaning that compared to ethanol, more phytochemicals were extracted as the polar solvent increased (i.e., more water content). This was attributed to the increased solubility of phytochemicals in water compared to ethanol. Protein and carbohydrates were found to be more soluble in water than ethanol (Hwang and Thi, 2014). Thus, the higher extraction yield in *Ba*A compared to *Ba*E, may be contributed by the higher amount of protein and carbohydrate in the *B. acmella* plant.

Phenolic compounds are important secondary metabolite groups present in plants and contribute significantly to the antioxidant activity in plants (Baba and Malik, 2015; Swallah et al., 2020). Thus, phenolic compounds can be used as a basis in screening antioxidant activity in plants. Flavonoids are the typical phenolics that contribute to antioxidant activity (Đudarić et al., 2015). They act by suppressing reactive oxygen formation, chelating trace elements involved in free-radical production, scavenging reactive species, and upregulating antioxidant defense (Ammar et al., 2009; El-Din and El-ahwany, 2016).

Folin-Ciocalteu (FC) and aluminum colorimetric methods were widely used to evaluate TPC and TFC. FC method is a fast, simple, inexpensive, robust, and reliable method to quantify phenolics in samples (Prior et al., 2005). Results from this study revealed that total phenolic and flavonoid contents were significantly influenced by the type of extraction solvent employed.

*Ba*E was found to have significantly higher TPC and TFC than *Ba*A. Similar findings were reported for *C. olitorius* (Ben Yakoub et al., 2018) and *Moringa oleifera* L. (Nobossé et al., 2018), indicating that ethanol extracts have higher TPC and TFC compared to aqueous extract. In contrast, Ismail et al. (2019) reported that the aqueous extract of *Adansonia digitata* has higher TPC and TFC than ethanol extract. Likewise, Gavamukulya et al. (2014), showed that *Annona muricata* leaves aqueous extract has a higher TPC than ethanol extract. The highest TPC and TFC in *Ba*E compared to previous studies are due to the characteristics of the phenolic compounds in *B. acmella* that are less hydrophilic compared to *Adansonia digitata* and *Annona muricata.*

There are numerous assays to evaluate the antioxidant activity of plant extracts. DPPH, ABTS, FRAP, ORAC, and CUPRIC (Dudonne et al., 2009; Esmaeili and Sonboli, 2010; Ozyurek et al., 2011; Moreno et al., 2020) are chemical screening methods used to evaluate the potential of extracts as an antioxidant agent. There is no pharmacological relevance using these chemical assays and no evidence for therapeutic health benefits. Different antioxidant methods produced diverse antioxidant activities due to the various mechanisms and reactions to the group of compounds present in plants (Floegel et al., 2011). The most widely used method of evaluating the antioxidant activity of plant extracts as potential antioxidant agents were DPPH, ABTS, and FRAP assays (Sridhar and Charles, 2019; Ilyasov et al., 2020).

DPPH and ABTS assay can scavenge radicals and reduce the redox-active compound by accepting an electron or hydrogen radical from antioxidants present in an extract to become a stable diamagnetic molecule. Meanwhile, FRAP assay can reduce non-stable ferric iron (Fe^3+^) to stable ferrous ion (Fe^2+^) by accepting an electron from antioxidants present in an extract and terminate the oxidation chain reaction (Bibi Sadeer et al., 2020).

Further evaluation on the antioxidant activity of *B. acmella* as a potential antioxidant agent was carried out in this study using the chemical screening method of DPPH, ABTS, and FRAP assays. L-ascorbic acid was chosen as a positive control. L-ascorbic acid is one of the synthesis antioxidants and has been widely used by researchers to study the antioxidant activity in plant extracts (Manssouri et al., 2020; Ho et al., 2021). A low IC_50_ value indicates the better antioxidant activity of the extracts as a potential antioxidant agent. The reaction between antioxidant and radical reagent caused the purple color of DPPH radical to turn to pale yellow (Prior et al., 2005), whereas the ABTS radical became blue to green color (Opitz et al., 2014). The color changes in DPPH and ABTS assays indicated the scavenging effects of the tested plant extract. In the ABTS assay, the radical cation was directly generated in a stable form using potassium persulfate before the treatment process to prevent the interference of compounds that will affect radical formation. Hence, the assay will become less susceptible to artifacts and prevents the overestimation of antioxidant capacity (Mzid et al., 2017). In the FRAP assay, the intense blue color complex was formed when the ferric tripyridyl triazine (Fe^3+^ TPTZ) complex was reduced to ferrous (Fe^2+^) by the action of electron-donating antioxidants (Benzie and Devaki, 2017).

Furthermore, the current findings showed that ethanol extract of *Ba*E (476.71 µg/ml) contributed to significantly lower IC_50_ values of DPPH assay than the aqueous extract of *Ba*A (860.67 µg/ml) (*p* < 0.05). This indicated that *Ba*E has higher antioxidant activity, at about 1.8 times higher than *Ba*A. Meanwhile, the ABTS assay showed no significant difference between *Ba*A (192.562 µg/ml) and *Ba*E (201.489 µg/ml), and a lower IC_50_ value compared to the DPPH assay. These findings were in line with a previous study by Izabela and Anna (Grzegorczyk-Karolak and Kiss, 2018) on antioxidant properties (DPPH and ABTS assays) of *Salvia virdis* L. shoots. They reported that based on the DPPH assay, ethanol extract contributed to significantly higher antioxidant activities than aqueous extract. No significant difference in the antioxidant activities of ethanol and aqueous extracts was found with ABTS assay. Similar patterns were also reported in studies on *Urtica urens* (Mzid et al., 2017)*, Harpagophytum procumbens* (Grabkowska et al., 2016) and *Rehmanniaglutinosa* (Piątczak et al., 2014).

ABTS assay is an aqueous-based assay that favors hydrophilic compounds, whereas DPPH assay is an organic (alcohol) and aqueous-based assay with an affinity for hydrophilic and hydrophobic compounds (Mzid et al., 2017; Grzegorczyk-Karolak and Kiss, 2018). Therefore, some highly water-soluble compounds in aqueous extracts may scavenge more ABTS radicals than DPPH radicals. This contributed to the lower IC_50_ value of *Ba*A in the ABTS assay (192.562 µg/ml) compared to the DPPH assay (476.71 µg/ml).

A further antioxidant test was carried out with the FRAP assay. The findings showed a similar pattern to the DPPH assay. Specifically, the antioxidant activity of *Ba*E (56.01 ± 6.46 mg L-ascorbic acid/g dry weight) was 17 times higher than *Ba*A (3.31 ± 0.53 mg L-ascorbic acid/g dry weight). The FRAP finding results corroborate a previous study which reported that non-oil seed legumes ethanol extracts had higher antioxidant activity than aqueous extracts (Diniyah et al., 2020).

### Correlation Between TPC and TFC to Antioxidant Activities (DPPH, ABTS, and FRAP) for *B. acmella* Extracts

The relationship between phenolic content (TPC and TFC) in *B. acmella* leaves extracts, and antioxidant activities (DPPH, ABTS, and FRAP scavenging activities) were analyzed by determining the Pearson’s correlations between them.

Results showed a significant positive correlation between TPC, TFC and antioxidant activities (FRAP, ABTS), while a significant negative correlation between TPC, TFC and DPPH activity of *B. acmella*.

A high negative correlation between TFC (R = −0.82) in *Prunus persica* L (Mokrani and Madani, 2016)*,* TPC (R = −0.791) in *Dittrichia viscosa* L. (Mssillou et al., 2021) and DPPH scavenging activity revealed that the lower TFC and TPC in plant extracts contributed to the higher DPPH radical scavenging activity. The present findings are in line with previous research showing negative correlations between TPC, TFC and DPPH scavenging activity in *B. acmella* extracts. TPC and TFC contribute to the high negative correlation (R = −0.869, R = − 0.995), which indicates that the lower the amount of TFC and TPC in *B. acmella* extracts, the lower the IC_50_ value, thus a better antioxidant potential. These results suggest that other compounds than phenolics and flavonoids play a key role in the inhibiting and trapping DPPH free radicals.

A high positive significant correlation was observed between TFC and antioxidant activity for ABTS (R = 0.718) and FRAP (R = 0.993) assays. This result corroborates the reports from previous studies. *Ginkgo biloba* extract showed positive correlations (R = 0.614) between TFC to FRAP activity (Sati et al., 2013) and *Morinda citrifolia* extract (R = 0.810) between TFC to ABTS activity (Thoo et al., 2010). Meanwhile, a high positive correlation was observed between TPC and FRAP (R = 0.881) and it was in line with a previous study in *Alphitonia philippinensis* extract (Ahmed et al., 2019). A high positive correlation indicated a high amount of TPC and TFC contributed to high FRAP value, thus a better antioxidant potential, suggesting that TPC and TFC plays a role in reducing ferric iron (Fe^3+^) to ferrous iron (Fe^2+^). The significantly positive correlations between TPC, TFC, ABTS, and FRAP in the present study suggested that the antioxidant activity of *B. acmella* leaves extracts was proportional to the concentration of phenolic and flavonoid compounds. Thus, it can be postulated that phenolic and flavonoid compounds contributed to the antioxidant activity of *B. acmella* extracts in ABTS and FRAP assays. This is due to the involvement of a single electron transfer mechanism in these three methods (Contreras-Calderón et al., 2011).

Further analysis was carried out on the relationship between antioxidant assays and negative correlations were detected between DPPH and ABTS (R = −0.686) and DPPH and FRAP (R = −0.985) in *B. acmella* extracts. These results are consistent with previous work by Thoo et al. (Thoo et al., 2010) on the *Morinda citrifolia* extract, which showed that there was a high negative correlation (R = −0.531) between DPPH and ABTS assays. The high negative correlation (R = −0.85) between DPPH and FRAP is in line with the findings of a study on *Prunus persica* L. extract (Mokrani and Madani, 2016). The current study indicated that some bioactive compounds in *B. acmella* have radical scavenging activity with ABTS and FRAP but not with DPPH assays.

In conclusion, phytochemicals will dissolve in the solvents depending on the polarity of solvents. The types of phytochemicals present in *B. acmella* extracts contributed to the activity in different antioxidant assays. High phenolic and flavonoid content in *B. acmella* contributed to the high antioxidant activity of ABTS and FRAP assays. Other compounds than phenolics and flavonoids contributed to the high antioxidant activity of the DPPH assay. Therefore, these finding results showed, the combination of group in plant secondary metabolites were contributes to the antioxidant activity in *B. acmella* extracts.

### Proliferation and Differentiation Activity of *B. acmella* Leaves Extracts on MC3T3-E1 Osteoblast Cells

Anabolic therapy is one of the approaches currently used to increase bone differentiation and bone formation in bone diseases such as osteoporosis. Therefore, a study was conducted on MC3T3-E1 osteoblast cells to determine if *B. acmella* leaves extracts have bone anabolic effects. Osteoblast cell growth is characterized by proliferation, differentiation, and mineralization of the matrix. MC3T3-E1 cell line was used in this study as a model to evaluate the effect of *B. acmella* leaves extracts on bone formation. These widely used cells differentiate into osteoblast-like cells and further develop into mature osteoblasts (Kim et al., 2014).

The MTT colorimetric assay is a widely used method to assess cellular metabolic activity in cell proliferation analysis. The principle is based on the reduction of 3-(4,5-dimethylthiazol-2-yl)-2,5-diphenyltetrazolium bromide by NAD(P)H-cellular oxidoreductase enzymes to insoluble formazan for the determination of viable cell number. Our results showed that *B. acmella* extracts could promote osteoblast proliferation, differentiation, and mineralization at appropriate concentrations of extracts. Figure 2B and Figure 3 showed that *Ba*E induced significantly higher cell proliferation compared to the control (*p* < 0.05) at the concentrations of 2.93 and 23.44 µg/ml on day 1. As for *Ba*A in Figure 2A, there were significantly higher cell proliferation than the control (*p* < 0.05) at the concentrations of 46.88 and 93.75 µg/ml on day 5, and concentrations of 5.8 µg/ml and 11.72 µg/ml on day 1. When compared, *Ba*E induced higher cell proliferation at lower concentrations and earlier days compared to *Ba*A. Therefore, concentrations of 2.93 µg/ml and 23.44 µg/ml for *Ba*E and 5.86 µg/ml and 11.72 µg/ml for *Ba*A were selected for further evaluation of the differentiation and mineralization activities of MC3T3-E1 cells.

Alkaline phosphatase (ALP) and collagen (COL1) are early osteogenic markers in matrix maturation and essential enzymes for osteoblast differentiation (Wu et al., 2017). Bone ALP, a glycoprotein found on osteoblast surfaces, reflects the biosynthetic activity of bone formation (Hyun et al., 2014; Wang et al., 2015; Luo et al., 2018). ALP induces and promotes mineralization in MC3T3-E1 cells after the formation of confluent monolayers (Thu et al., 2017). It is released into the osteoid to initiate minerals deposition (Lee and Choi, 2006). Mineral depositions indicate bone formation activity by deposition of calcium in the bone nodules (Ahmad et al., 2018). As osteoblasts differentiation is severely compromised in osteoporosis (Zhang et al., 2018), the promotion of osteoblast differentiation is an effective strategy to prevent pathological bone loss.

The present findings showed that the proliferation (141.32%) and ALP activity (66.91 nmol/well pNPP) were highest at *Ba*E concentration of 2.93 µg/ml while collagen percentage (196.81%) was highest at *Ba*E concentration of 23.44 µg/ml. No significant difference (*p* > 0.05) was found between the ALP activity of the two concentrations of *Ba*E 2.93 µg/ml and 23.44 µg/ml. This indicated that both extracts showed the most significant contribute to the highest ALP activity and it was correlated with collagen production. Similar findings were reported in a previous study, showing ALP activity correlation with collagen production of MC3T3-E1 cells after treatment with *Eurycoma longifolia* (Thu et al., 2017). The relationship between collagen production and ALP activity was contributed by the interactions between collagens matrix and integrin receptors (Thu et al., 2017). Results from this study suggested that treatment with *Ba*E may promote interactions between collagen matrix and integrin receptors in MC3T3-E1 cells. Calcium deposition was detectable in MC3T3-E1 cells during the first 14 days of culture cells. The highest mineralization was found in BaE_23.44 (208.39%) and BaE_2.93 (204.57%) with no significant different (*p* > 0.05) was found between them.

In the present finding, ALP activity in *Ba*E was more than 200% higher than control on day 14. This ALP activity was higher compared to that of *B. acmella* leaves of Widyowati (2011), which was 169% higher than control. The different solvent polarities used during extraction may have contributed to different phytochemicals being extracted. According to Lamien-Meda et al. (2010) (Lamien-Meda et al., 2010), different locations, climatic conditions, altitudes, and temperatures exposed by the plants may also influence the synthesis of phytochemicals. Thus, the different ALP activity in the present findings compared to Widyowati (2011) may be due to the different solvent extraction used and the different location or environment of the *B. acmella* plantation. These factors may contribute to phytochemical variation and abundance in *B. acmella* plant.

In summary, both *B. acmella* extracts contributed to the early and late differentiation activity of MC3T3-E1 cells. Ethanol extract (*Ba*E) produced higher osteoblast differentiation compared to aqueous extract (*Ba*A). The highest collagen content were observed at the *Ba*E concentrations of 23.44 µg/ml, respectively. The highest ALP activity and mineral depositions were observed at the *Ba*E concentrations of 2.93 µg/ml and 23.44 µg/ml, respectively with no significant different between both concentrations. Thus, the findings from this study indicated that both *Ba*E concentrations promoted higher cell proliferation, differentiation, and mineralization than *Ba*A. It can be deduced that *Ba*E could promote osteoblast cell proliferation, differentiation, and mineralization for new bone formation. Further study is needed to isolate the chemical compounds present in the most competent solvent extract (*Ba*E) that might have contributed to the antioxidant and bone anabolic activities.

### Relationship Between TPC and TFC to Anabolic Activities (Collagen, ALP and Mineralization) of *B. acmella* Extracts

The relationship between phenolic content (TPC, TFC) and antioxidant activities (DPPH, FRAP, ABTS) in *B. acmella* leaves extracts and anabolic activities (Collagen, ALP and Mineralization) were analyzed by determining the Pearson’s correlations between them.

A high significant positive correlation was found between TFC and FRAP assay to cell proliferation, collagen, ALP and mineralization activity, between TPC to ALP and mineralization activity, and ABTS assay to collagen and mineralization activity. A moderate significant positive correlation was found between TPC to cell proliferation and collagen activity and ABTS assay to cell proliferation and ALP activity. Meanwhile, a highly significant negative correlation was found between DPPH to cell proliferation, collagen, ALP and mineralization activity. The findings from this study were supported by previous studies. Flavonoid compounds of rutin isolated from *Chrozophora tinctoria* (Abdel-Naim et al., 2018) and phenolic compounds of paradol isolated from *Aframomum meleguta* enhances bone cells proliferation and ossification (Abdel-Naim et al., 2017). A review by Torre (2017) reported that bone anabolic activity is related to the phenolic compound due to their antioxidant properties.

### Phytochemical Compounds Identified With Antioxidant and Bone Anabolic Activities of *B. acmella* Leaves Ethanol Extract

GCMS and LCMS are versatile tools for separation, quantification, quantitation, and identification of unknown compounds. It provides excellent separation of compounds, fast method and is capable of producing high-quality chemical fingerprints and determination of compounds, qualitatively, and quantitatively. These could be useful for elucidating the relationship between compounds present in plants and their pharmacological effects (Liang et al., 2004; Değirmenci and Erkurt, 2020). GCMS is suitable for non-targeted metabolite profiling of volatile and thermally stable non-polar or derivatized polar metabolites as well as targeted analysis of derivatized primary metabolites. Meanwhile LCMS is suitable for non-volatile, polar or thermally labile (Tugizimana et al., 2013). Therefore, GCMS and LCTOFMS were used to determine the compounds that may contribute to the antioxidant and bone anabolic activities of *Ba*E.

Supplementary Tables S1, S2 showed the compound present in the *B. acmella*. There are new compounds detected in the present study compared to the review in previous studies (Abdul Rahim et al., 2021) and it has been identified in other plants. The difference in the phytochemical present in the present study cultivated in Malaysia compared to the previous study cultivated over the world was due to the different environmental conditions. A review by Tohidi et al. (2019) reported that the number and compound presented in *Thymus* genus were different in different locations.

GCMS analysis shows a high abundance of terpenoids in *Ba*E which contributed to the antioxidant activity confirmed the higher antioxidant activity in the ABTS compared to the DPPH assay employed in this study. This is consistent with a previous study, where *P. ecuadorense*, which is rich in sesquiterpene and monoterpene, produced better antioxidant activity in the ABTS assay (Valarezo et al., 2020). Moreover, the ABTS method has been demonstrated to be suitable for assessing lipophilic antioxidants (Andrade et al., 2017). Hence, the lower antioxidant activity in the DPPH assay in this study could also be explained by the inability of terpenoids to donate a hydrogen atom. Terpenoids have low solubility in the reaction medium of the DPPH assay, which uses ethanol as the solvent (Mata et al., 2007).

Phenolics includes flavonoids are significant plant compounds that have prominent antioxidant activity due to their hydroxyl group. The total phenolic, flavonoid, and antioxidant activity (ABTS and FRAP) and anabolic activity (cell proliferation, collagen, ALP, mineralization) of *B. acmella* extracts were significantly and positively correlated. Therefore, the present of flavonoids using LCTOFMS in *Ba*E contributed to the antioxidant and anabolic activities. The high levels of terpenoids in GCMS analysis, which have antioxidant activity close to that of flavonoid constituents, break free-radical chain reactions, and caused their irreversible oxidation into inert compounds (Değirmenci and Erkurt, 2020).

Meanwhile, other compounds including alkylamide, fatty acid ester, alkene, alkane, coumarine, alkaloid and purine nucleosides in *Ba*E demonstrated a significantly negative correlation between the antioxidant activity (DPPH) and anabolic activity and that of the total phenolic and flavonoid content.

Natural antioxidative molecules act synergistically against free radicals (Lu and Foo, 2001; Mokrani and Madani, 2016). Therefore, the antioxidant and anabolic potential of the studied *Ba*E may be attributed to the presence of terpenoids (α-cubebene, caryophyllene, caryophyllene oxide and phytol) and flavonoids (apigenin and pinostrobin). A synergism between compounds in *Ba*E may contribute to antioxidant and anabolic activities.

## Conclusion

In summary, this study is the first attempt to demonstrate the bone anabolic effects of *B. acmella* aqueous and ethanol exracts by the proliferation, differentiation, and mineralization activities on osteoblasts. The ethanol extract was superior to aqueous extracts, as it was believed to contain an abundance of phytochemicals with antioxidant and bone anabolic activities. This was proven by the different antioxidant activities found in DPPH, ABTS, and FRAP antioxidant assays, with positive and negative correlations. The current results revealed that terpenoids, alkyl amides, fatty acid esters, alkene, alkane, coumarin, alkaloid and purine nucleosides in *Ba*E were considered to contribute to antioxidant activities in *Ba*E and bone anabolic effects of the *Ba*E extract. Among the identified compounds in *Ba*E, terpenoids of α-cubebene, caryophyllene, caryophyllene oxide, phytol and flavonoids of pinostrobin and apigenin are the compounds that may contribute to both antioxidant and anabolic activities.

Therefore, the current results showed that *Ba*E leaves have the potential to be developed further as antioxidant and anti-osteoporosis agents. Further studies need to be performed to isolate, characterized, and elucidate the specific compounds and underlying mechanisms responsible for these antioxidant and anabolic activities.

## Data Availability

The original contributions presented in the study are included in the article/Supplementary Material, further inquiries can be directed to the corresponding authors.
