# Supplementary material for: Phytochemical Analysis, Antioxidant and Bone Anabolic Effects of Blainvillea acmella (L.) Philipson
Source: Front Pharmacol. 2022 Jan 17;12:796509. doi: 10.3389/fphar.2021.796509 (PMC8802550; doi:10.3389/fphar.2021.796509)
Supplement: Supplementary file 1 [file DataSheet1.docx]

Supplementary Material

# Supplementary Table

Supplementary Table S1: Biological activity of known compounds present in *Ba*E using GCMS

| **Bil/ Number** | **Retention time**  **(min)** | **Area (%)** | **Compound name** | **Molecular weight**  **(g/mol)** | **Molecular**  **formula** | **Group** | **Activity** | **References** |
| --- | --- | --- | --- | --- | --- | --- | --- | --- |
| 1 | 29.4573 | 0.0397 | α-Cubebene | 204.35 | C_15_H_24_ | Terpenoid | - Antioxidant - DPPH assay in *Piper miniatum* - Anabolic - Compound in herbal of Ee-Zhi-Wan Stimulate osteoblast proliferation and increased ALP activity on osteoblast cell | (Salleh, Kammil, Ahmad, & Sirat, 2015)  (Yan-Bin Wu, 2012) |
| 2 | 30.7043 | 0.4864 | (E)-2-Tetradecene | 196.377 | C_14_H_28_ | Alkene | - Antioxidant - DPPH and ABTS assay in *Leonutus leonurus* | (Tonisi et al., 2020) |
| 3 | 31.4864 | 5.3406 | Caryophyllene | 204.357 | C_15_H_24_ | Terpenoid | - Antioxidant   - DPPH and FRAP assay in *Aquilaria crassna,*   - DPPH assay in *Piper miniatum Blume;*   *Phlomis bourgaei* Boiss;  *Annona salzmanii* and *A. pickelii* | (Dahham et al., 2015)  (Costa et al., 2011; Sarikurkcu, Sabih Ozer, Cakir, Eskici, & Mete, 2013) |
|  |  |  |  |  |  |  | - Anabolic   - - Compound in *Atractylodes japonica* stimulated osteoblast proliferation and differentiation by increase collagen content, cell proliferation, ALP activity and calcium depositions in MC3T3-E1 cells     - Compound promoted osteoblastic mineralization in bone marrow cell cultures | (Chang, Choi, & Kim, 2014)  (Yamaguchi & Levy, 2016) |
| 4 | 31.9936 | 0.0845 | 2-isopropyl-5-methyl-9-methylene-Bicyclo [4.4.0] dec-1-ene | 204.35 | C_15_H_24_ | Terpenoid | - Antioxidant - DPPH and ABTS assay in *Oleo europaea* - DPPH assay in *Pulicaria arabica* | (Adegborioye et al., 2018)  (Djermane, Gherraf, Arhab, Zellagui, & Rebbas, 2016) |
| 5 | 32.7123 | 0.0234 | 1,2,3,4,4a,5,6,8a-octahydro-7-methyl-4-methylene-1-(1-methylethyl), (1α.,4aα.,8aα.)- naphthalene | 204.35 | C_15_H_24_ | Terpenoid | - Anabolic - Compound in herbal of Ee-Zhi-Wan Stimulate osteoblast proliferation and increased ALP activity on osteoblast cell | (Yan-Bin Wu, 2012) |
| 6 | 33.0927 | 0.3823 | Z,Z,Z-1,4,7,-Cycloundecatriene, 1,5,9,9-tetramethyl | 204.35 | C_15_H_24_ | Alkene | - Anabolic - Compound in *Acmella oleracea* promoted cell growth on dental pulp stem cell | (Samsurrijal, Rahim, Azizan, Noor, & Vuanghao, 2019) |
| 7 | 34.4455 | 2.225 | Germacrene D-d3 | 207.37 | C_15_H_21_D_3_ | Terpenoid | - Antimicrobial | (Sun et al., 2015) |
| 8 | 34.7202 | 0.2857 | 4-(2,6,6-trimethyl-1-cyclohexen-1-yl)- 3-Buten-2-one | 206.32 | C_14_H_22_O | Terpenoid | - Antioxidant - DPPH assay in *Pulicaria arabica* | (Djermane et al., 2016) |
| 9 | 35.1852 | 0.1513 | Bicyclogermacrene | 204.35 | C_15_H_24_ | Terpenoid | - Antioxidant - DPPH assay in *Annona salzmanii* and *A. pickelii.* | (Costa et al., 2011) |
| 10 | 35.5445 | 1.9661 | 1-Pentadecene | 210.398 | C_15_H_30_ | Alkene | - Anabolic - Compound in *Eclipta prostrata* stimulated the proliferation and increased ALP activity on primary osteoblast cells | (Lin et al., 2010) |
| 11 | 36.2843 | 2.2194 | 2,4-Di-tert-butylphenol | 206.323 | C_14_H_22_O | Phenolic | - Antioxidant - DPPH assay | (Varsha et al., 2015; Yoon et al., 2006) |
| 12 | 38.9475 | 1.3009 | Caryophyllene oxide | 220.35 | C_15_H_24_O | Terpenoid | - Antioxidant - DCFHDA on human skin fibroblast with *T. vulagare* extract - DPPH assay in *Piper miniatum* - Anabolic - Compound in herbal of Ee-Zhi-Wan stimulate osteoblast proliferation and increased ALP activity on osteoblast cell | (Coté, Boucher, Pichette, & Legault, 2017)  (Salleh, Kammil, et al., 2015)  (Yan-Bin Wu, 2012) |
| 13 | 40.1523 | 0.9663 | 1-Hexadecene | 224.42 | C_16_H_32_ | Alkene | - Anabolic - Compound in herbal of Ee-Zhi-Wan stimulate osteoblast proliferation and increased ALP activity on osteoblast cell | (Yan-Bin Wu, 2012) |
| 14 | 48.7125 | 0.472 | 1-Octadecene | 252.5 | C_18_H_36_ | Alkene | - Antioxidant – DPPH assay in *Chlamydomonas reinhardtii* CC | (Renukadevi, Saravana, & Angayarkanni, 2011) |
| 15 | 51.1643 | 0.1411 | Phthalic ester, diisobutyl ester | 278.34 | C_16_H_22_O_4_ | Fatty acid ester | - Antibacterial - Antimicrobial | (Naik et al., 2020) |
| 16 | 52.4536 | 29.534 | N-Isobutyl-2(E),6(Z),8(E)-decatrienamide / Spilanthol | 221.33 | C_14_H_23_NO | Alkaloid | - Antioxidant - FRAP assay in *Acmella* *oleracea* Murr. (Asteraceae) - DPPH assay in *Acemalla uliginosa* (Sw.) Cass | (Abeysiri, Dharmadasa, Abeysinghe, & Samarasinghe, 2013)  (Maimulyanti, Prihadi, & Safrudin, 2016) |
| 17 | 53.8909 | 0.3338 | Hexadecanoic acid, methyl ester | 270.45 | C_17_H_34_O | Fatty acid ester | - Antioxidant - DPPH assay in *D. esculentum* - Antimicrobial | (Naik et al., 2020) |
| 18 | 54.7998 | 0.5463 | Dibutyl phthalate | 278.34 | C_16_H_22_O_4_ | Fatty acid ester | - Antibacterial | (Mini Shobi & Gowdu Viswanathan, 2018) |
| 19 | 55.3282 | 3.3819 | α-Springene | 272.46 | C_20_H_32_ | Terpenoid | - Antibacterial | (Nurjanah, Sudaryanto, Widyasanti, & Pratiwi, 2016) |
| 20 | 56.4907 | 3.3944 | 19-Hydroxy-13-epimanoyl oxide | 306.48 | C_20_H_34_O_2_ | Terpenoid | - Anticancer | (Li et al., 2016) |
| 21 | 59.9148 | 0.1868 | 9,12-Octadecadienoic acid, methyl ester, (E,E)- | 294.472 | C_19_H_34_O | Fatty acid ester | - Anabolic - Compound in *Acmella oleracea* promoted cell growth on dental pulp stem cell - Compound in herbal of Ee-Zhi-Wan stimulate osteoblast proliferation and increased ALP activity on osteoblast cell | (Samsurrijal et al., 2019)  (Yan-Bin Wu, 2012) |
| 22 | 60.1261 | 0.3691 | (Z,Z,Z)-9,12,15-Octadecatrienoic acid, methyl ester/ Linolenic acid methyl ester | 292.456 | C_19_H_32_O | Fatty acid ester | - Anabolic - Compound in *Acmella oleracea* promoted cell growth on dental pulp stem cell | (Samsurrijal et al., 2019) |
| 23 | 60.6968 | 10.975 | Phytol | 128.17 | C_20_H_40_O | Terpenoid | - Antioxidant - DPPH and ABTS assay in *Leonutus leonurus;*   *Oleo europea;*   - DPPH assay in *D. esculentum* - Anabolic - Compound in *Acmella oleracea* promoted cell growth on dental pulp stem cell - Compound in *Eclipta prostrata* stimulated the proliferation and increased ALP activity on primary osteoblast cells | (Tonisi et al., 2020)  (Naik et al., 2020)  (Samsurrijal et al., 2019)  (Lin et al., 2010) |
| 24 | 61.5423 | 1.3939 | N-Isobutylundeca-(2E,4E)-diene-8,10-diynamide | 229.32 | C_15_H_19_NO | Alkylamide | - Antioxidant - DPPH assay in *Acemalla uliginosa* (Sw.) Cass | (Maimulyanti et al., 2016) |
| 25 | 62.5146 | 2.0269 | 9,12,15-Octadecatrienoic acid, ethyl ester, (Z,Z,Z)- | 306.48 | C_20_H_34_O_2_ | Fatty acid ester | - Antioxidant - DPPH, ABTS and FRAP assay in *P. cognatum* extract. | (Eruygur et al., 2020) |
| 26 | 66.8475 | 0.3417 | (7S)-trans-bicyclo[4.3.0]-3-nonen-7-ol | 138.21 | C_9_H_14_O | Terpenoid | - Antioxidant - DPPH and FRAP assay in *Dictamnus angustifolius.* | (Sun et al., 2015) |
| 27 | 69.8911 | 0.1263 | Octadecanoic acid, butyl ester | 340.5836 | C_22_H_44_O | Fatty acid ester | - Antioxidant - DPPH and FRAP assay in *L. Ciliata* | (Akshatha, Prakash, & Nalini, 2016) |
| 28 | 70.187 | 4.1615 | N-(2-Phenylethyl)(2E,6Z,8E)-decatrienamide | 269.38 | C_18_H_23_NO | Alkylamide | - Antioxidant -DPPH assay in *Acemalla uliginosa* (Sw.) Cass | (Maimulyanti et al., 2016) |
| 29 | 73.5055 | 0.3075 | Eicosane | 282.5 | C_20_H_42_ | Alkane | - Antioxidant -DPPH and ABTS assay in *Leonutus leonurus* | (Tonisi et al., 2020) |
| 30 | 74.4566 | 0.317 | 1,2-Benzenedicarboxylic acid, diisooctylester | 390.56 | C_24_H_38_O_4_ | Fatty acid ester | - Anabolic - Compound in herbal of Ee-Zhi-Wan stimulate osteoblast proliferation and increased ALP activity on osteoblast cell | (Yan-Bin Wu, 2012) |

Supplementary Table S2: Biological activity of known compounds present in *Ba*E using LCTOFMS

| Name/  Bil Number | Retention time  (min) | % Area | m/z  [M+H] ^+^ [M+K] ^+^  [M+Na] ^+^  [M+NH4] ^+^ | Molecular weight (g/mol) | Compound name | Molecular formula | Group | Activity | Reference |
| --- | --- | --- | --- | --- | --- | --- | --- | --- | --- |
| 1 | 24.578 | 0.7874 | 354.2158 | 336.4 | Methyl (1R,18R)-17-ethyl-3,13-diazapentacyclo [3.3.1.02,10.04,9.013,18] nonadeca-2(10),4(9),5,7,16-pentaene-1-carboxylate / Catharanthine | C_21_H_24_N_2_O_2_ | Alkaloid | - Antioxidant - Anticancer | (Moon et al., 2018) |
| 2 | 25.013 | 0.2574 | 355.2000 | 354.4 | Yohimbine | C_21_H_26_N_2_O_3_ | Alkaloid | - Aphrodisiac | (Morales, 2000) |
| 3 | 25.001 | 0.8025 | 354.1885 | 315.4 | (2E,4E)-N- [2-(4- hydroxyphenyl) ethyl] dodeca-2,4-dienamide | C_20_H_29_NO_2_ | Alkylamide | NA | NA |
| 4 | 52.222 | 2.5781 | 274.2140 | 251.41 | (2E,4E)-N-(2-methylpropyl) dodeca-2,4-dienamide | C_16_H_29_NO | Alkylamide | - Antimicrobial | (Sharma & Arumugam, 2021) |
| 5 | 5.364 | 0.1361 | 207.0968 | 184.24 | 3-methyl-6-(2-methyl propyl) piperazine-2,5-dione | C_9_H_16_N_2_O_2_ | Amino acid | NA | NA |
| 6 | 39.288 | 2.1575 | 278.1463 | 260.29 | Cyclo (tyrosyl-prolyl) | C_14_H_16_N_2_O_3_ | Amino acid | - Antimicrobial | (Kilian, Tshanga, Oidu, & Milne, 2011) |
| 7 | 36.339 | 0.0806 | 223.0829 | 222.19 | Reticulol | C_11_H_10_O_5_ | Coumarin | - Moderate antioxidant activity against DPPH radicals | (Sritharan, Savitri Kumar, Jayasinghe, Araya, & Fujimoto, 2019) |
| 8 | 41.595 | 1.3671 | 335.2161 | 312.4 | (9Z,12E)-15,16-dihydroxyoctadeca-9,12-dienoic acid | C_18_H_32_O_4_ | Fatty acid | NA | NA |
| 9 | 42.841 | 0.0317 | 289.2053 | 288.38 | 2-decyl-3-hydroxypentanedioic acid | C_15_H_28_O_5_ | Fatty acid | NA | NA |
| 10 | 40.114 | 1.6694 | 260.1833 | 242.31 | (Z)-2-octylpent-2-enedioic acid | C_13_H_22_O_4_ | Fatty acid | NA | NA |
| 11 | 40.768 | 1.8777 | 244.1563 | 243.3 | 8-acetamido-2-methyl-7-oxononanoic acid | C_12_H_21_NO_4_ | Fatty acid | NA | NA |
| 12 | 40.727 | 0.0016 | 539.2761 | 500.6 | Mupirocin | C_26_H_44_O_9_ | Fatty acid | - Antimicrobial | (Khoshnood et al., 2019) |
| 13 | 37.786 | 0.0711 | 285.1790 | 284.31 | Alpinetin Methyl Ether | C_17_H_16_O_4_ | Flavonoid | - Antibacterial | (Salleh, Ahmad, & Yen, 2015) |
| 14 | 36.1 | 2.0619 | 271.16956 | 270.28 | Pinostrobin | C_16_H_14_O_4_ | Flavonoid | - Antioxidant– Compound *in Lamium album* contributed to FRAP, ABTS, DPPH. - Anabolic- Promoted proliferation, differentiation and mineralization of MC3T3-E1 cells | (Uwineza, Gramza-Michałowska, Bryła, & Waśkiewicz, 2021)  (Gu, Fu, Yuan, & Liu, 2017; Saah, Siriwan, & Trisonthi, 2021) |
| 15 | 40.944 | 2.2624 | 271.18555 | 270.24 | Apigenin | C_15_H_10_O_5_ | Flavonoid | - Antioxidant – FRAP and ABTS assay - Anabolic activity-   Increased alkaline phosphatase (ALP) and mineralization activity in human mesenchymal stem cells (hMSCs) | (Tian et al., 2021)  (Xue Zhang et al., 2015) |
| 16 | 25.013 | 0.1540 | 354.17029 | 336.381 | 9-methoxy-4,4-dimethyl-13-phenyl-6,14-dioxatetracyclo [8.4.0.0^2,7^.0^3,5^] tetradeca-1,7,9-trien-11-one | C_21_H_20_O_4_ | Flavonoid | NA | NA |
| 17 | 4.005 | 0.2134 | 294.13892 | 276.284 | (2S)-4-hydroxy-2-(2-hydroxypropan-2-yl)-7-methyl-2,3-dihydrofuro[3,2-g] chromen-5-one | C_15_H_16_O_5_ | Furanochromones | NA | NA |
| 18 | 43.075 | 1.0821 | 249.20264 | 248.32 | Pechueloic Acid/  Rupestonic acid | C_15_H_20_O_3_ | Guianese | Antiviral - Inhibited replication of influenza virus infection through activation of heme oxygenase 1 | (Yin et al., 2017) |
| 19 | 38.176 | 0.4288 | 263.20483 | 262.4 | 3-methoxy-2-(3-methylbut-2-enyl)-5-pentylphenol | C_17_H_26_O_2_ | Methoxyphenols | NA | NA |
| 20 | 40.693 | 0.0098 | 270.1347 |  | Methyl 3,4,5-trimethoxycinnamate | C_13_H_16_O_5_ | Phenylpropanoid | Anti-inflammatory activity in both RAW264.7 macrophages and in a macrophage adipocyte co-culture | (Olajide, Akande, da Silva Maia Bezerra Filho, Lepiarz-Raba, & de Sousa, 2020) |
| 21 | 35.194 | 0.5047 | 268.10254 | 267.24 | Adenosine | C_10_H_13_N_5_O_4_ | Purine nucleosides | Antioxidant and antiproliferative activity on the HepG2 cells | (Lu, Li, Qiao, Qiu, & Liu, 2017) |
| 22 | 34.966 | 0.2267 | 276.11633 | 253.29 | N-[1-(4-methoxy-6-oxopyran-2-yl)-2-methylbutyl] acetamide | C_13_H_19_NO_4_ | Pyranones and derivatives | NA | NA |
| 23 | 36.305 | 0.0553 | 275.18445 | 274.35 | (4aS)-6,7-dihydroxy-1,1,4a-trimethyl-3,4,10,10a-tetrahydro-2H-phenanthren-9-one/ Nimbidiol | C_17_H_22_O_3_ | Terpenoids | Potent intestinal disaccharidase and glucoamylase inhibitor for diabetes treatment | (Mukherjee & Sengupta, 2013) |
| 24 | 39.863 | 0.0258 | 301.21008 | 300.4 | Dehydroabietic acid | C_20_H_28_O_2_ | Terpenoids | Anti-inflammatory activity at the transcriptional level in results from NF-κB- or AP-1-mediated luciferase assays in macrophage cell line | (Kim et al., 2019) |
| 25 | 41.225 | 1.0896 | 224.20456 | 206.32 | Carylophyllene Oxide | C_14_H_22_O | Terpenoids | - Antioxidant - DCFHDA on human skin fibroblast with *T. vulagare* extract - DPPH assay in *Piper miniatum* - Anabolic - Compound in herbal of Ee-Zhi-Wan stimulate osteoblast proliferation and increased ALP activity on osteoblast cell | (Coté et al., 2017)  (Salleh, Kammil, et al., 2015)  (Yan-Bin Wu, 2012) |
| 26 | 35.194 | 0.2669 | 269.12561 | 246.302 | (3aS,5aS,9bR)-5a,9-dimethyl-3-methylidene-3a,4,6,7,8,9b-hexahydrobenzo[g][1] benzofuran-2,5-dione | C_15_H_18_O_3_ | Terpenoids | NA | NA |
| 27 | 36.026 | 0.1614 | 231.14062 | 230.3 | Dehydrocostus lactone | C_15_H_18_O_2_ | Terpenoids | Antioxidant activity -Protective effect of dehydrocostus lactone against osteoblast damage induced by antimycin A | (Seo & Choi, 2012) |
| 28 | 38.277 | 0.0769 | 443.28156 | 420.6 | (1S,4S,5R,9S,10R,13R,14R)-14-hydroxy-5,9-dimethyl-14-{[(3-methylbutanoyl) oxy] methyl} tetracyclo [11.2.1.0^1,10^.0^4,9^]hexadecane-5-carboxylic acid | C_25_H_40_O_5_ | Terpenoids | NA | NA |
| 29 | 41.414 | 0.0070 | 335.18958 | 334.4 | [(1S,3aR,5R,5aR,8aR,9S,9aR)-1,5,8a-trimethyl-2,8-dioxo-3a,4,5,5a,9,9a-hexahydro-1H-azuleno[6,5-b] furan-9-yl] 2-methylpropanoate /  Arnicolide C | C_19_H_26_O_5_ | Terpenoids | Antiviral against influenza A virus *in vitro* | (Xiaoli Zhang et al., 2018) |
| 30 | 40.991 | 0.0143 | 237.18268 | 23.35 | (4aR,5S,6S,8R,8aS)-6-hydroxy-3,8-dimethyl-5-propan-2-yl-4a,5,6,7,8,8a-hexahydro-1H-naphthalen-2-one / Petasitolone | C_15_H_24_O_2_ | Terpenoids | NA | NA |
| 31 | 31.107 | 0.0390 | 284.1911 | 266.33 | (4aR,5S)-9,9a-dihydroxy-3,4a,5-trimethyl-5,6,7,8,8a,9-hexahydro-4H-benzo[f1] benzofuran-2-one | C_15_H_22_O_4_ | Terpenoids | NA | NA |
| 32 | 2.453 | 0.9389 | 192.06027 | 169.18 | Pyridoxine / Vitamin B6 | C_8_H_11_NO_3_ | Vitamin B | Anticancer | (Wei & Mao, 2020) |
